# Epigenetic variability in cells of normal cytology is associated with the risk of future morphological transformation

**DOI:** 10.1186/gm323

**Published:** 2012-03-27

**Authors:** Andrew E Teschendorff, Allison Jones, Heidi Fiegl, Alexandra Sargent, Joanna J Zhuang, Henry C Kitchener, Martin Widschwendter

**Affiliations:** 1 Statistical Genomics Group, Paul O'Gorman Building, UCL Cancer Institute, University College London, 72 Huntley Street, London WC1E 6BT, UK; 2 Department of Women's Cancer, UCL Elizabeth Garrett Anderson Institute for Women's Health, University College London, 74 Huntley Street, London WC1E 6AU, UK; 3 Department of Gynecology and Obstetrics, Innsbruck Medical University, Anichstrasse 35, A-6020 Innsbruck, Austria; 4 School of Cancer and Imaging Science, Manchester Academic Health Science Centre, Central Manchester University Hospitals NHS Foundation Trust, Oxford Road, Manchester M13 9WL, UK

## Abstract

**Background:**

Recently, it has been proposed that epigenetic variation may contribute to the risk of complex genetic diseases like cancer. We aimed to demonstrate that epigenetic changes in normal cells, collected years in advance of the first signs of morphological transformation, can predict the risk of such transformation.

**Methods:**

We analyzed DNA methylation (DNAm) profiles of over 27,000 CpGs in cytologically normal cells of the uterine cervix from 152 women in a prospective nested case-control study. We used statistics based on differential variability to identify CpGs associated with the risk of transformation and a novel statistical algorithm called EVORA (Epigenetic Variable Outliers for Risk prediction Analysis) to make predictions.

**Results:**

We observed many CpGs that were differentially variable between women who developed a non-invasive cervical neoplasia within 3 years of sample collection and those that remained disease-free. These CpGs exhibited heterogeneous outlier methylation profiles and overlapped strongly with CpGs undergoing age-associated DNA methylation changes in normal tissue. Using EVORA, we demonstrate that the risk of cervical neoplasia can be predicted in blind test sets (AUC = 0.66 (0.58 to 0.75)), and that assessment of DNAm variability allows more reliable identification of risk-associated CpGs than statistics based on differences in mean methylation levels. In independent data, EVORA showed high sensitivity and specificity to detect pre-invasive neoplasia and cervical cancer (AUC = 0.93 (0.86 to 1) and AUC = 1, respectively).

**Conclusions:**

We demonstrate that the risk of neoplastic transformation can be predicted from DNA methylation profiles in the morphologically normal cell of origin of an epithelial cancer. Having profiled only 0.1% of CpGs in the human genome, studies of wider coverage are likely to yield improved predictive and diagnostic models with the accuracy needed for clinical application.

**Trial registration:**

The ARTISTIC trial is registered with the International Standard Randomised Controlled Trial Number ISRCTN25417821.

## Background

It has been proposed that epigenetic variation may contribute to the risk of complex genetic diseases like cancer and that differential exposure to environmental risk factors may underlie much of this variation [1,2]. Consistent with this view, a recent study has shown that regions that are differentially methylated between normal and cancer tissue appear to be highly variable in cancer itself, and that identification of cancer-relevant markers may therefore benefit from statistics that measure differential variability [3]. Based on these insights, we here aimed to demonstrate that analysis of epigenetic variability in prospectively collected normal cells can predict the risk of future morphological transformation.

In order to demonstrate this in humans, two requirements are mandatory: the cells that are used for epigenetic analyses need to be (i) the cells of origin for the studied cancer, and (ii) they need to be collected years in advance of the onset of cytological and morphological signs of cancer. Currently, the only human organ that meets these two requirements is the uterine cervix. Thus, we used Illumina Infinium technology [4] to measure DNA methylation (DNAm) at 27,578 CpG sites in cytologically entire normal cells (liquid-based cytology (LBC) samples) from the uterine cervix of 152 women (aged 19 to 55 years) in a nested prospective case-control study within ARTISTIC (A Randomised Trial of HPV Testing in Primary Cervical Screening [5,6]). Prospective cases were women who developed a cervical intraepithelial neoplasia of grade 2 or higher (CIN2+) within 3 years of sample draw, while controls were women who remained disease-free. To further support our data we used completely independent LBC samples with abnormal cytology and associated controls, as well as cervical cancer tissue and normal cervix specimens.

## Methods

### Study population

#### The ARTISTIC trial

The LBC samples we analyzed were collected from women as part of the ARTISTIC trial [5,6]. All women underwent two screening rounds with an interval of 3 years. Within the ARTISTIC trial, women, aged 19 to 64 years who were undergoing routine screening as part of the English National Health Service Cervical Screening Programme in Greater Manchester were randomly assigned in a ratio of 3:1 to either combined LBC and human papilloma virus (HPV) testing where the results were revealed and acted on, or to combined LBC and HPV testing where the HPV result was concealed from the patient and investigator. There were a total of 24,510 eligible women at entry. In the first round of screening 453 women had CIN2+. In the second round of screening 75 women (who were screen-negative in the first round and who had a sample stored from the first round) had developed CIN2+ (44 were HPV-positive and 31 were HPV-negative in the first round). Seventy-seven women who had not developed any cytological changes were matched (age and HPV status in round 1) to the cases. The cytologically normal samples from round 1 from these 152 women were used for DNAm analysis. Further details, cytology and HPV scoring are described in Additional file 1. This trial is registered with the International Standard Randomised Controlled Trial Number ISRCTN25417821.

#### DNA methylation nested case control study

A total of 152 samples in a prospective nested case control study within ARTISTIC were selected for DNAm analysis. Cases were 75 women who had normal cytology in screening round 1 but demonstrated CIN2+ after 3 years in round 2. Controls were 77 women who had normal cytology at entry and in the second screening round. Cases and controls were matched for age (Wilcox rank sum test *P *= 0.95) and HPV status: 92 were HPV-positive (44 cases and 48 controls) and 60 were HPV-negative (31 cases and 29 controls) at entry (Fisher test *P *= 0.74). Informed consent was obtained from the main study population and this study has been approved by the ethical committee (National Research Ethics Service Reference Number 10/H1107/15).

#### Other DNA methylation data sets

In addition to the nested case control prospective study within ARTISTIC, we used two additional DNAm data sets that were also generated using the same Illumina Infinium 27 k platform. Set 1 comprised a total of 30 LBC samples (19 HPV-negatives and 11 HPV-positives) with normal cytology and 18 LBC samples (all HPV-positive) with CIN2+, as described in [7]. Set 2 comprised a total of 63 cervical tissue samples: 48 cervical cancers, 15 normals. The normal cervical tissue samples were from women (mean age 55.4 years) who underwent a hysterectomy for fibroids of the uterine corpus, that is, these women did not have fibroids in the uterine cervix. The 48 cervical cancer specimens were from women (matched for age with mean age 56.8 years) who were treated at the Innsbruck Medical University. Of these 48 cancers, 26 were of stage 1, 10 of stage 2, 4 of stage 3 and 7 of stage 4 (1 sample had missing stage information). In terms of grade, 7 were of grade 1, 28 of grade 2 and 11 of grade 3 (2 samples had no grading). All specimens were obtained with informed consent and approval from the ethics committee UN4044-290/4.9.

#### DNA extraction and methylation assay

The DNA extraction protocol is described in Additional file 1. Genome-wide methylation analysis using the Illumina Infinium Methylation27K beadchip (Illumina Inc., USA, WG-311-1201) was performed. This chip interrogates the methylation status of over 27,000 CpG sites throughout the human genome, covering the promoters of over 14,000 genes [4]. Further details are in Additional file 1.

#### Data availability

All data in this manuscript have been deposited in the Gene Expression Omnibus repository [8] under accession number [GSE30760].

#### mRNA expression data of normal cervical and cervical cancer tissue

We used the publicly available normalized expression data (all Affymetrix) from the Gene Expression Omnibus with accession numbers [GSE9750] [9], [GSE7803] [10], and [GSE6791] [11]. For each downloaded data set we only selected the normal squamous cervix epithelial and squamous cervical cell carcinoma samples: for [GSE9750] there were 24 normal and 33 cancers, for [GSE7803] there were 10 normals and 21 cancers, and for [GSE6791] there were 8 normals and 20 cancers. Probes mapping to the same Entrez ID were averaged, resulting in 13,213 genes [GSE9750], 13,262 genes [GSE7803] and 20,827 genes [GSE6791].

#### mRNA expression data analysis

From the above three expression data sets we built an integrated (merged) expression set over 42 normal cervical epithelial samples and 74 cervical cancers using a procedure that we have validated many times previously [12-14]. Briefly, there were 13,213 genes in common between all three expression arrays. For each of these genes and for each expression set we then renormalized the gene expression profile to mean zero and unit variance, yielding 'z-profiles'. For each gene, its z-profile in each of the three studies was then merged. This resulted in a merged expression set over 13,213 genes and 116 samples (42 normal and 74 cancers). For each gene, we then computed a *t*-statistic *P*-value against normal/cancer status. Of the 140 risk genes, 86 were found in the merged expression set. Of these 86, 46 exhibited differential expression *P*-values < 0.05. A binomial test was used to test the significance of the skew towards differential under- or overexpression. To adjust for any global (that is, over all 13,213 genes) skew towards under- or overexpression, we also estimated the *P*-value using a Monte Carlo procedure (1,000 Monte Carlo runs) in which 86 genes were selected at random and a binomial test *P*-value was recomputed. The fraction of the 1,000 runs with a binomial test *P*-value more extreme than the observed gives an independent *P*-value estimate.

### Statistical methods

Full details of statistical methods are in Additional file 1. Brief descriptions of the different parts of the statistical analysis are given below.

#### Quality control and inter-array normalization

The raw DNAm data were subject to a similar quality control procedure as used in our previous publication [7].

#### Supervised analyses

To identify CpGs associated with age (aCpGs) we used surrogate variable analysis [15]. False discovery rate (FDR) estimation was implemented as in the q-value package [16]. To identify age-independent variable CpGs (vCpGs), we adjusted the data for age, and subsequently estimated the variances for each CpG. Because of the heteroscedasticity of β-values [17], we also estimated the variance using *R*-values (defined as *R = M/U*) [18]. To identify differentially variable CpGs (DVCs) between prospective CIN2+ cases and normals, we used Bartlett's test [19]. In doing so, variances were estimated after the methylation profiles were linearly adjusted for age within each phenotype.

#### EVORA: Epigenetic Variable Outliers for Risk Prediction Analysis

Full details and the model assumptions on which EVORA is based are described in Additional file 1. There are three important statistical aspects to EVORA: (i) identification of candidate risk CpGs; (ii) identification of samples that constitute methylation outliers; and (iii) a method for assigning risk to each sample, which is robust and independent of the scale used. For (i) we use Bartlett's test [19], since our hypothesis is that DVCs defined by outlier profiles are more likely to define risk CpGs [3]. To define outliers in a scale-independent fashion (ii) we use the COPA (Cancer Outlier Profile Analysis) transformation [20]. Lastly, to assign a risk score to a sample, we use an adaptive index algorithm framework [21]. EVORA is freely available as an R-package (*evora*) [22].

## Results

### DNA methylation variability is increased in cytologically normal cells predisposed to neoplasia

**Figure 1:**
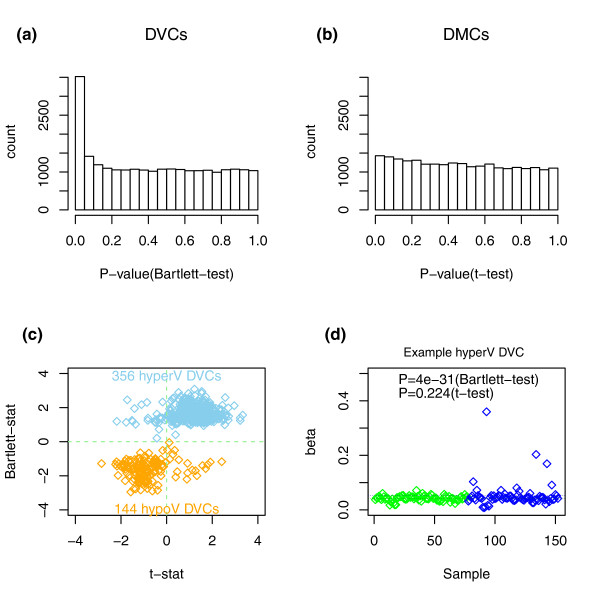
**Differentially variable and differentially methylated CpGs**. **(a) **Histograms of *P*-values derived from Bartlett's test comparing differences in variance between normal samples that become neoplastic (CIN2+) and samples that remain normal (differentially variable CpGs (DVCs)). **(b) **Histograms of *P*-values derived from *t*-tests comparing differences in mean CpG methylation levels between the same two phenotypes (differentially methylated CpGs (DMCs)). **(c) **Scatterplot of Bartlett statistics (logarithm of the ratio of the variance in prospective CIN2+ to that in normal) shown on the y-axis against the corresponding *t*-statistics (x-axis) for the top 500 DVCs. The numbers of hypervariable (hyperV) and hypovariable (hypoV) DVCs are given. **(d) **Typical methylation profile of a hypervariable DVC (blue = prospective CIN2+, green = normal). The thin dashed lines indicate the mean levels of methylation in each phenotype. The *P*-values shown are from a Bartlett's test (differential variability) and *t*-test (differential methylation).

A stringent quality control and inter-array normalization procedure resulted in a normalized data matrix of methylation values (β-values, 0 < β < 1) over 24,039 CpGs and 152 samples (Methods). Prospective cases (*n *= 75) and controls (*n *= 77) were matched for age and HPV status (Methods). Following the suggestion that epigenetic variability may mark genes that contribute to the risk of cancer [1,3,23], we hypothesized that differential variability in normal tissue might be associated with an increased risk of neoplasia. We thus derived a list of CpGs that showed significantly different (age-adjusted) variability between future (CIN2+) cases and controls (DVCs) (Methods). We observed many DVCs (Figure 1a) and among the top 500 (FDR < 0.0001; Additional file 2) the majority (73%) were more variable in future cases (Figure 1c). The set of DVCs was largely unchanged if variability was not adjusted for age or if also adjusted for HPV status (Additional file 3). In contrast, testing for differential methylation using *t*-statistics (differentially methylated CpGs (DMCs)) did not yield genome-wide significance levels (FDR ~0.6 for the top 50 CpGs; Figure 1b). DVCs that showed significantly higher variance in future cases (hypervariable DVCs) generally exhibited small yet consistent increases in mean methylation levels (Figure 1d; Additional file 2). Inspection of typical methylation profiles of such DVCs revealed that the increased or decreased variability was due to approximately 20 to 30% changes in DNAm present in only a relatively small number of 'outlier' samples (Figure 1d; Additional file 4). Developmental genes, and in particular polycomb group targets (PCGTs) [24] (see Additional file 1 for precise definition), were enriched only among the DVCs showing increased variance in the normal cells of prospective cancer cases (odds ratio = 4.9 (3.9 to 6.3), *P *< 1*e*-31) and were six times more likely to exhibit higher variability in prospective cases than lower (Additional file 5). PCGTs were also the most highly enriched gene category out of a total of 6,173 gene sets in a Gene Set Enrichment Analysis [25] (Additional file 6). Random permutation of sample labels also showed that this enrichment could not have arisen by chance (Additional file 5). Thus, all these results indicate that increased DNAm variability affects PCGTs and is an intrinsic property of normal cells predisposed to neoplasia.

### Age-associated variation in DNA methylation also correlates with the risk of neoplasia

An unsupervised singular value decomposition and a supervised linear regression analysis (adjusted for CIN2+ status) both confirmed a strong age-associated signature (644 CpGs at FDR < 0.05) with the majority (392, 61%) hypermethylated with age (Additional files 7 and 8). Interestingly, we observed that the 644 CpGs undergoing age-associated changes in DNAm (aCpGs) showed specific directional changes associated with the future development of CIN2+ even though these changes were not individually significant (Additional file 9). Specifically, those CpGs undergoing significant hypermethylation with age were generally also hypermethylated in future CIN2+ cases (Additional file 9). To test further if age-hypermethylated aCpGs are indeed associated with CIN2+, we compared their methylation levels in an independent data set (also generated with the Illumina 27 k platform) consisting of 30 normal and 18 age-matched CIN2+ samples (Set 1, Methods) [7]. The mean methylation level of these aCpGs was also significantly higher in the CIN2+ samples of this set (Additional file 10).

### Significant overlap of hypervariable DVCs and age-hypermethylated aCpGs

**Figure 2:**
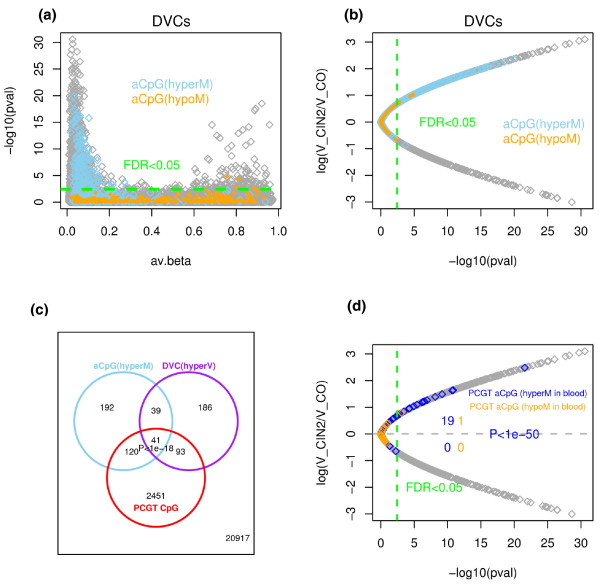
**Relation between differentially variable and age-associated CpGs**. **(a) **Bartlett test *P*-values (on -log10 scale) of CpGs indicating significance of differential variability (between prospective CIN2+ and controls) (y-axis) versus their average β-value across all samples (x-axis). CpGs undergoing age-associated (aCpG) hypermethylation (hyperM) or hypomethylation (hypoM) are colored as indicated. **(b) **The ratio (on log scale) of variability in prospective CIN2+ to variability in controls (y-axis) versus significance level (x-axis). Skyblue (orange) denotes CpGs significantly hypermethylated (hypomethylated) with age (aCpGs) in normal cells from uterine cervix. The green dashed line represents the FDR cutoff value of 0.05 for calling DVCs. **(c) **Venn diagram illustrating overlaps of age-hypermethylated CpGs with DVCs that are hypervariable (hyperV) in prospective CIN2+, and with PCGT CpGs. A total of 41 CpGs overlapped between all three categories and 20,917 CpGs were in none of the three categories. The *P*-value (estimated from a multiple binomial test) indicates the random chance of observing 41 or more overlapping CpGs. **(d) **As (b) but now highlighting the 68 and 20 CpGs that map to PCGTs and undergo age-associated hyper- (blue) and hypomethylation (red) in whole blood samples [7]. Among these CpGs, we give the number that are significantly differentially variable (FDR < 0.05, green dashed line) and their distribution in terms of increased or decreased variance in future CIN2+ cases. *P*-value is from a binomial test.

Next, we explored the relationship between DVCs and aCpGs. Many CpGs showing age-associated hypermethylation also showed significant increases in (age-adjusted) variability within the normal tissues of future CIN2+ cases, while age hypomethylated aCpGs did not (Figures 2a-c). Thus, CpGs that are more variable in prospective CIN2+ cases independently of age overlapped significantly with CpGs that undergo age-associated hypermethylation in normal tissue independently of prospective disease status. This could mean that detecting DNAm changes across a group of individuals of the same age but who may have had variable lifetime exposures to environmental risk factors (and therefore be at variable disease risk) is similar to detecting age-associated changes in a population of differently aged individuals (since lifetime exposures accumulate with age). Because PCGTs were enriched in both hypermethylated (hyperM) aCpGs and hypervariable (hyperV) DVCs (Figure 2c), it was natural to ask if aCpGs mapping to PCGTs and that had been identified from other tissues (for example, whole blood) [7] would also exhibit a preferential skew towards hypervariability. Remarkably, out of the 69 PCGT CpGs identified as hypermethylated with age in whole blood [7], the overwhelming majority were more variable in the epithelial cells of future CIN2+ cases (Figure 2d). In contrast, the 20 PCGT CpGs undergoing age-associated hypomethylation in blood showed no skew towards either increased or decreased variability (Figure 2d). Thus, we can conclude that genes prone to epigenetic variation are also prone to undergo age-associated hypermethylation and that PCGTs define a significant subset of these genes.

### Differentially hypervariable CpGs predict risk of intraepithelial cervical neoplasia

**Figure 3:**
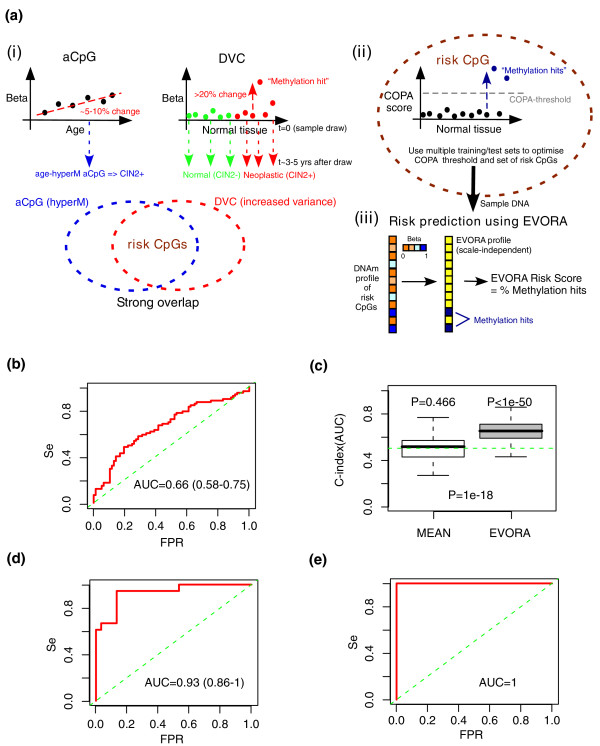
**Epigenetic variable outliers for risk prediction analysis**. **(a) **Flowchart describing the EVORA model. (i) Age-associated DNAm variation and age-independent differentially variable DNAm are both correlated with the risk of prospective neoplasia (CIN2+). aCpGs undergoing age-associated hypermethylation (hyperM) overlap strongly with differential variable CpGs (DVCs) that exhibit increased variance in future CIN2+ cases. This overlap defines, for a given training set, the pool of candidate risk CpGs. (ii) Multiple training/test set partitions in a ten-fold internal cross-validation on COPA-transformed (Methods) methylation profiles is used to optimize the COPA threshold and the set of risk CpGs. (iii) Risk prediction using EVORA: for an independent sample its risk score is estimated as the fraction of risk CpGs with a β-value larger than the optimal threshold, as evaluated in the COPA-basis. **(b) **EVORA receiver operating characteristic (ROC) curve, AUC and its 95% confidence interval in the ARTISTIC cohort (152 normal samples: 75 future CIN2+, 77 normals). **(c) **Comparison of C-index (AUC) values obtained using EVORA with a classification algorithm based on detecting differences in mean methylation levels (mean) in the ARTISTIC cohort. Boxplots are over 100 distinct training-test set partitions and *P*-values are from a Wilcoxon test detecting deviation from the expected null (C-index = 0.5) as well as between the two classification algorithms. **(d) **EVORA ROC curve in set 1 (48 liquid-based cytology samples: 18 CIN2+, 30 normals). **(e) **EVORA ROC curve in set 2 (63 cervical tissue samples: 48 cancers, 15 normals). In all ROC curves, AUC values and 95% confidence intervals shown. FPR: false positive rate; Se: sensitivity.

Based on these results, we proposed the following model in which epigenetic variance may be used to predict the risk of neoplastic transformation. Since the typical DVC methylation profile (Figure 1d) is one in which a small number of samples exhibit increased outlier methylation (≥ 20% methylation change), we hypothesized that cancer-risk in a given sample may be associated with the number of such risk CpGs (hypervariable DVCs/hypermethylated aCpGs) becoming 'methylation hits' (Figure 3a). To test this idea, we applied a novel statistical algorithm called EVORA (Methods; Figure 3a), which aims to assess the risk of neoplastic transformation from the number of methylation outliers. Using multiple training/test set partitions, we found that EVORA could predict the future risk of CIN2+ in blind test sets (area under the curve (AUC) = 0.66 (0.58 to 0.75), *P *< 0.05; Figure 3b), while an analogous classifier based on differences in mean methylation levels could not (AUC = 0.51 (0.30 to 0.72), *P *= 0.46; Figure 3c).

### Risk CpGs identified in normal cells can detect intraepithelial neoplasia and cervical cancer

**Figure 4:**
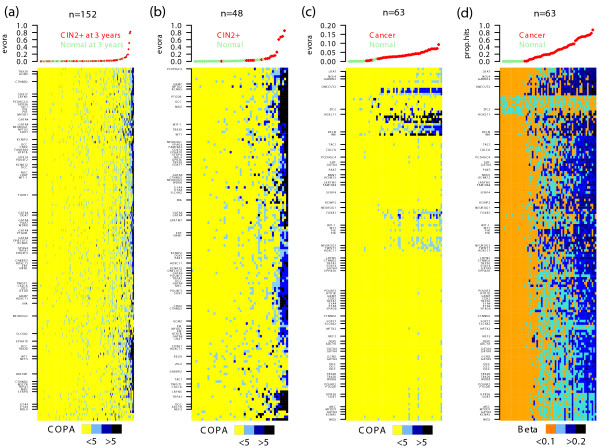
**Heatmaps over risk CpGs**. **(a-c) **Heatmaps of COPA-transformed methylation values for the top 140 risk CpGs that are (i) significantly hypermethylated with age and (ii) show significant increased variability in future CIN2+ cases, as determined in the ARTISTIC cohort. Color codes for COPA scores: yellow = COPA score < 1 (no methylation); skyblue = COPA score < 5. Outliers denoted by blue = methylation COPA score > 5 and black = methylation COPA score > 10. CpGs have been hierarchically clustered using a Manhattan distance metric. Those mapping to PCGTs are labeled with their associated gene. Samples have been ordered according to their EVORA risk score as shown in the panels above heatmaps. (a) ARTISTIC cohort: 152 samples (75 prospective CIN2+ (red), 77 no CIN2+ at last follow-up (green). (b) Set 1: 48 samples (18 CIN2+ (red), 30 normals (green)). (c) Set 2: 63 cervical tissue samples (48 cancers (red), 15 normals (green)). (d) Heatmap depicts the same data matrix as in (c) but with the methylation values on the β-value scale where CpG β-values have been median normalized to zero. The corresponding scores now depict the percentage of methylation hits as measured on the beta-scale.

**Figure 5:**
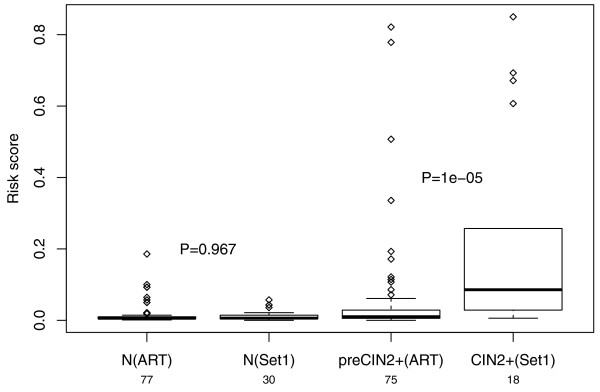
**Cross-comparison of EVORA risk scores**. Boxplots of EVORA risk scores (y-axis) of the 77 normal LBC samples in ARTISTIC (N(ART)), the 30 normal LBC samples of set 1 (N(Set1)), the 75 prospective CIN2+ LBC samples in ARTISTIC (preCIN2+(ART)), and the 18 CIN2+ samples of set 1 (CIN2+ (Set1)). Wilcox-test *P*-values between N(ART) and N(Set1), and between preCIN2+(ART) and CIN2+(Set1) are given.

**Figure 6:**
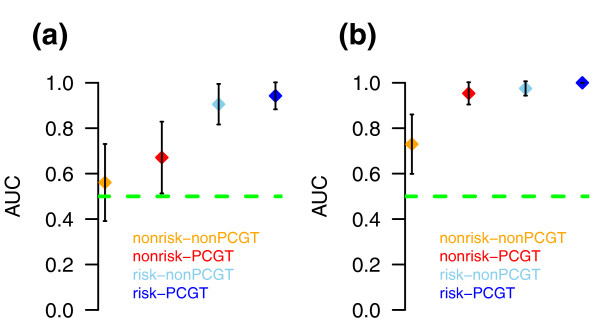
**EVORA AUC values as a function of differential variability and PCGT status**. **(a, b) **Comparison of EVORA AUC measures for four different CpG classes in (a) set 1 consisting of 18 CIN2+ LBC samples and 30 normal LBC samples, and in (b) set 2 consisting of 48 cervical cancers and 15 cervical normal tissues. Of the 140 risk CpGs, 69 mapped to PCGTs (risk-PCGT class) and 71 did not (risk-nonPCGT class). In addition, we randomly selected 70 non-differentially variable, non-age-associated CpGs (nonrisk) that mapped and that did not map to PCGTs. The random selection was done 100 times and AUC values averaged. Also provided are 95% confidence intervals.

EVORA identified a total of 140 risk CpGs (hypervariable DVCs and hypermethylated aCpGs; Figure 4a; Additional file 11), of which 69 mapped to PCGTs. We postulated that this pool of 140 risk CpGs would also necessarily diagnose CIN2+ status, since for established neoplastic cells we would expect an even higher fraction of these CpGs to be hypermethylated. Indeed, in an independent Illumina Infinium 27 K methylation data set of normal cervical smears and age-matched CIN2+ samples (set 1; Methods), EVORA was able to predict CIN2+ status with very high accuracy (Figures 3d and 4b). Importantly, while the risk scores of the normal samples in ARTISTIC and set 1 were comparable to each other, the scores of the CIN2+ samples were significantly higher than those of normal cells that only become CIN2+ within 3 years (Figure 5), reflecting a progressive increase from normal cells at low risk, to normal cells at high risk, and finally to cells in a pre-invasive neoplastic state. Importantly, risk CpGs (that is, differentially variable CpGs) identified from the ARTISTIC cohort predicted CIN2+ status better than CpGs that were not differentially variable, even if they mapped to PCGTs (Figure 6). Since risk CpGs performed similarly irrespective of PCGT status (Figure 6), this indicates that differential variability is the key feature of cells at risk of morphological transformation and not PCGT status.

Next, we explored the methylation profiles of the 140 risk CpGs in an independent set of cervical cancers and corresponding age-matched normal tissue (set 2; Methods). The outlier risk scores were significantly reduced (Figure 4c) owing to the fact that risk CpGs exhibited much more of a bi-modal methylation pattern between cancer and normal tissue; that is, risk CpGs were invariably either hemi- or fully methylated across a high proportion of the cancers (Figure 4d). Nevertheless, a small subset of risk CpGs retained their outlier profiles in cervical cancer and EVORA was therefore still highly predictive of cancer status (Figure 4c). Adapting EVORA to the original beta methylation scale to better capture the observed bi-modal methylation profiles yielded a perfect discrimination of normal and cancer tissue (Figures 3e and 4d).

### Risk genes are underexpressed in cervical cancer

To evaluate if the genes associated with the identified risk CpGs show expression changes in cervical cancer, we built an integrated data set of mRNA expression profiles over 13,213 genes and encompassing 42 normal cervical epithelial and 74 cervical cancer (squamous cell carcinoma) specimens (Methods). The integration was done using a renormalization procedure that we have validated previously [12-14]. Of the 140 risk genes, 86 could be mapped to this merged data set. Comparison of average mRNA levels of our 86 risk genes between normal and cancer tissue confirmed that risk genes showed lower average expression levels in cervical cancer (Additional file 12). Analysis of individual gene statistics further showed that 46 were differentially expressed and that there was a significant skew towards underexpression in cancer (Additional file 12). Moreover, in only 13 of 1,000 random selections of 86 gene sets (Monte Carlo analysis described in Methods) did we observe a skew as significant as the one observed for the risk genes (Additional file 12).

## Discussion

In this work we have addressed two fundamental questions. First, do DNAm changes precede the morphological signs of neoplastic transformation? And second, can these epigenetic changes (a) predict the risk of neoplastic development, and (b) allow detection of early (non-invasive) cancers? We have addressed these questions in the context of the uterine cervix, currently the only human organ allowing relatively easy access to the cell of origin of the associated cancer well in advance of the first morphological signs of neoplastic transformation.

Based on the hypothesis that epigenetic variability may reflect exposure to various genetic and non-genetic risk factors (including age), and that differential epigenetic variability may reflect underlying differential exposure to these factors [1,3], we posited that differentially variable methylated CpGs (DVCs) might be potential risk indicators of neoplastic transformation. Our prediction algorithm based on epigenetic variance (EVORA; Figure 3A) could predict, with low but statistically significant accuracy (AUC = 0.66, *P *< 0.05), prospective CIN2+ status in blind test sets (Figure 3b). In contrast, an analogous classifier that uses differential methylation instead of differential variability statistics could not predict prospective CIN2+ status (Figure 3c). Using EVORA, the set of risk CpGs could also accurately identify independent CIN2+ and cervical cancer samples (AUC > 0.9; Figures 3d, e and 4). Using these additional data sets, we also showed that differential variability is a more important characteristic of risk CpGs (supporting the findings in [3]) than PCGT status (Figure 6). Thus, these results support the view that differential variability is a key characteristic of cells predisposed to morphological transformation.

We have obtained these results in the context of cervical cancer using a study design that was matched for age and HPV status. Since HPV infection is a well-known necessary causal factor for cervical cancer [26], it is thus reasonable to assume that those HPV-negative samples that later developed a CIN2+ will have done so only by acquiring an HPV infection after sample collection [27]. It is important, therefore, to point out that the increased DNAm variability at the identified loci only increases the risk of CIN2+ in the background of an existing HPV infection. Given that HPV infection is a necessary but not sufficient factor to cause cervical carcinogenesis (most HPV-infected women do not develop cervical cancer), it is indeed very likely that unknown 'tumor-suppressor' factors play a role in determining which HPV-infected women develop a CIN2+. Thus, our data support a model in which increased DNAm variability at the identified loci may compromise specific tumor suppressor functions, predisposing HPV-infected women to develop a CIN2+. In this context, it is also important to note that we did not observe HPV infection to have a major effect on DNAm patterns and the EVORA risk score itself was not correlated with HPV status (Additional file 13). Nevertheless, we did observe a very weak marginal association of DNAm with HPV status within only the prospective CIN2+ sample subgroup (Additional file 13). Thus, while increased DNAm variability may synergize with a background HPV infection to increase the risk of CIN2+, HPV infection itself only appears to have a minor effect on DNAm patterns in the earliest stages of cervical carcinogenesis. It is possible, however, that HPV infection may cause significant DNAm and mRNA changes during the latter stages of carcinogenesis, as reported, for instance, in the case of head and neck cancers [11,28,29].

In line with our hypothesis that epigenetic variable CpGs are involved in carcinogenesis, we find that they become more homogeneously methylated (that is, either hemi- or fully methylated) across cancers (Figure 4d), in stark contrast to their heterogeneous (that is, unmethylated versus hemi- or fully methylated) outlier profiles in potent CIN2+ and established CIN2+ cells (Figure 4a, b). This transition from heterogeneous outlier profiles in the earliest stages of cervical carcinogenesis to more homogeneous methylation profiles in the more advanced cancer stage points towards a dynamic process in which DNAm is initially acquired in a largely stochastic manner, as hypothesized in [1], but that later becomes more homogeneous and deterministic. However, we should point out that risk CpGs remain highly variably methylated across the invasive cancers, as previously reported in [3].

Interestingly, the heatmaps of the prospective and pre-invasive cancer series (Figures 4a, b) also revealed the existence of a striking bi-modality within the cases only, with a small subset of cases exhibiting a highly correlated pattern of CpG methylation, in contrast to the more stochastic pattern of methylation seen in the majority of samples. It is particularly interesting that the risk CpGs identified in ARTISTIC generated this same characteristic stochastic versus correlated bi-modal pattern in the CIN2+ samples of set 1. To investigate this further, we used an unsupervised clustering algorithm to divide up the CIN2+ samples (of set 1) and the cervical cancers (of set 2) each into two clusters according to the average level of methylation over all CpGs mapping to CpG islands excluding the 140 risk CpGs (Additional file 14). Interestingly, in both sets, the EVORA risk scores obtained from the 140 riskCpGs were significantly higher for the cases exhibiting the higher global levels of CpG island methylation (Additional file 14). Thus, the risk CpGs appear to correlate particularly strongly with CIN2+ samples and cervical cancers of overall higher CpG island methylation levels. It will be interesting, therefore, to explore the relationship, if any, of the identified risk CpGs to a potential CpG island methylator phenotype (CIMP) [30,31] in cervical cancer. Using an unsupervised consensus clustering over the 1,000 most variable CpGs in the ARTISTIC set - that is, the unsupervised procedure used in [31,32] to define a CIMP, did not yield a clear CIMP, nor did the clusters correlate with the EVORA risk scores, suggesting that there is no CIMP (in the sense defined in [31,32]) present three years in advance of morphological transformation (Additional file 15).

Over the years extensive evidence has accumulated to demonstrate that PCGT methylation is crucially involved in carcinogenesis [33-37]. Although we have here provided substantial evidence that differential variability is a more fundamental characteristic of transformation than PCGT status (Figure 6), our analyses nevertheless implicate a subset of PCGTs in the earliest stages of carcinogenesis (69 out of the 140 risk CpGs mapped to PCGTs). Numerous risk CpGs were also annotated to genes that have been previously demonstrated to be linked to cervical cancer, including *CALCA *[38], *CXCR4 *[39], *DCC *[40], *HOXC10 *[10], *MYOD1 *[41], *SFRP4 *[42], *SOX1 *[43], *TWIST1 *[44] and *WT1 *[45]. However, we note that only *HOXC10 *had its risk CpG site located within 200 bp upstream of the transcription start site. In fact, of the 140 risk CpG sites, only 64 were within 200 bp of the start site, and of these only 24 were upstream. Thus, even though we observed the genes associated with our risk CpGs to be frequently underexpressed in cervical cancer, it remains to be seen if methylation of the identified risk CpG sites plays a part in the underlying regulatory mechanism.

Our data also revealed a remarkably strong overlap between CpGs undergoing age-associated hypermethylation in normal tissue and CpGs that were hypervariable in prospective CIN2+ cases. PCGTs made a large component of this overlap and age-associated hypermethylated CpGs also correlated significantly with CIN2+ status, further strengthening our previous findings [7]. We stress that even those PCGT CpGs that were identified as undergoing age-associated hypermethylation in other tissues (for example, whole blood) [7] were also more hypervariable in prospective CIN2+ samples and could indicate the risk of neoplastic transformation. This strong overlap between hypermethylated aCpGs and hypervariable DVCs is consistent with a model in which differential exposure to risk factors accumulates with age, thus generating the observed stochastic epigenetic variability. That these methylation changes and overlaps are not due to changes in cell-type composition is supported by many studies [7,46-48]. We also observed here that changes in cell-type composition were more likely to be captured by age-independent variable CpGs (vCpGs), defined as CpGs that showed the largest (age-adjusted) variation across all normal samples. Specifically, these maximally varying vCpGs exhibited fairly large (> 80%) changes in methylation, did not overlap with DVCs, and accordingly were neither enriched for PCGTs nor discriminatory of prospective CIN2+ status (Additional file 9). In fact, vCpGs were enriched for Gene Ontology terms related to extracellular space and mesenchymal features, including also many cell differentiation markers (Additional file 16), and thus it is possible that methylation variation in these CpGs reflects variations in the epithelial to mesenchymal and stromal cell ratio. Since risk CpGs were not enriched for these stromal and mesenchymal features, it supports the view that their observed methylation changes reflect clonal heterogeneity within the epithelial cell population only.

The reliability of the methylation data generated from the ARTISTIC cohort is strongly supported by emerging biology and successful validations in two independent data sets. Nevertheless, to further check the reliability of the Infinium methylation data, we compared the β-values to those obtained using Methylight. Matched Infinium-Methylight data were available for *SOX1 *and *WT1 *in 48 LBC samples (set 1) [49]. We found statistically significant agreement between the two data types for both *SOX1 *and *WT1 *(Additional file 17).

Unfortunately, the limited coverage of the Infinium 27 K array (only 0.1% of the CpGs present in the human genome [50]) means that we could not fully explore the spatial methylation patterns around the identified risk CpG sites. As shown in Hansen *et al. *[3] in the context of cancer, differential variability is associated with increased spatial variability and loss of stability of the sharp methylation boundaries. Thus, it will be interesting to explore the spatial variability around the identified CpG risk sites with a more comprehensive and unbiased technology such as the Infinium 450 K methylation beadarray [51], as this may reveal a similar loss of methylation boundaries surrounding these sites.

## Conclusions

We have demonstrated that variability in DNAm, which is associated with age and other factors, and which occurs well in advance (at least 3 years) of the first morphological neoplastic changes, is associated with the risk of neoplastic transformation. More generally, we have demonstrated that inter-individual epigenetic variance is an intrinsic characteristic of cells that become neoplastic.

## Abbreviations

aCpG: age-associated CpG; ARTISTIC: A Randomised Trial of HPV Testing in Primary Cervical Screening; AUC: area under the curve; bp: base pair; CIMP: CpG island methylator phenotype; CIN2+: cervical intraepithelial neoplasia of grade 2 or higher; COPA: Cancer Outlier Profile Analysis; DMC: differentially methylated CpG; DNAm: DNA methylation; DVC: differentially variable CpG; EVORA: Epigenetic Variable Outliers for Risk Prediction Analysis; FDR: false discovery rate; HPV: human papilloma virus; LBC: liquid-based cytology; PCGT: polycomb group target; vCpG: variable CpG.

## Competing interests

The authors declare that they have no competing interests.

## Authors' contributions

AET designed and performed the statistical analyses. MW designed the study. HCK and AS contributed samples. AJ helped with processing of the samples. JZ helped with statistical analyses. IJ helped with funding. AET and MW wrote the manuscript. All authors have read and approved the manuscript for publication.

## Supplementary Material

Additional file 1 **Supplementary information with further details of Materials and methods**. Click here for file

Additional file 2 **The top 500 differentially variable CpGs (DVCs)**. We provide the Illumina probe ID, Entrez ID, gene symbol, the ratio of age-adjusted variance of future CIN2+ cases to controls, *P*-value from Bartlett's test, corresponding q-value, and for comparison also the difference in mean methylation levels, *t*-statistic and corresponding *t*-test *P*-values. Click here for file

Additional file 3 **Scatterplots of the Bartlett test b-statistics**. Scatterplots of the Bartlett test b-statistics (that is, log2(ratio of variances of prospective CIN2+ to normal)) obtained without adjustment of age or HPV status (b), against the corresponding ones obtained after adjustment for age (b(adj.Age)), after adjustment for HPV status (b(adj.HPV)), and after adjustment for both age and HPV status(b(Adj.Age+HPV)). Click here for file

Additional file 4 **Methylation profiles of 10 of the top hypervariable DVCs in the ARTISTIC cohort (all 10 shown are also among the 140 risk CpGs)**. Green denotes normal samples and blue denotes prospective CIN2+ cases. Click here for file

Additional file 5 **PCGT enrichment odds ratio (OR) among the top 500 DVCs and the corresponding relative odds ratio (ROR) of PCGT enrichment (ROR = OR(more variable in future CIN2+)/OR(less variable in future CIN2+))**. Also shown is the expected ROR for the null case where the top 500 CpGs were selected after a random permutation of sample labels. The *P*-value reflects the significance of the difference between the observed and null ROR. Click here for file

Additional file 6 **Gene Set Enrichment Analysis using a one-tailed Fisher's exact test of the top 500 differentially variable CpGs (DVCs), done separately for those with increased variance in future CIN2+ cases, and those with decreased variance in future CIN2+ cases**. The Molecular Signatures Database (MSigDB, Broad Institute) [25] was used. The columns label the gene list, the number of genes in the list, the number represented on the 27 K array, the corresponding fraction, the number that overlap with the top 500 vCpGs, the *P*-value of enrichment, the adjusted *P*-value, and the gene symbols for the enriched genes. Click here for file

Additional file 7 **The 644 age-associated CpGs (FDR < 0.05)**. We provide the Illumina probe ID, Entrez ID, gene symbol, *t*-statistic and *P*-value from linear regression against age, and estimated q-value (FDR). Click here for file

Additional file 8 **Heatmap of *P*-values of association between the singular vectors of a singular value decomposition on the inter-array normalized adjusted data, with experimental (Bisulfite conversion efficiency controls (BSCE) 1 and 2, beadchip) and phenotypic factors (CIN2+ status, HPV status and age)**. *P*-values were estimated using *t*-tests (CIN2+ and HPV status), linear regression (age and BSCE BSCE: bi-sulfite conversion efficiency) and ANOVA (beadchip). Color codes: *P *< 1*e*-10 (dark red), *P *< 1*e*-5 (red), *P *< 0.001 (orange), *P *< 0.05 (pink), *P *> 0.05 (white). Click here for file

Additional file 9 **Age-associated CpGs and variable CpGs and their relation to CIN2+ status**. **(a) **Scatterplot of *t*-statistics of the 644 age-associated CpGs (FDR < 0.05). Their *t*-statistics relative to CIN2+ status (y-axis) are plotted against their age associated *t*-statistics (x-axis). Colored CpGs denote the 175 age-PCGT CpGs (skyblue = age-hypermethylated; orange = age-hypomethylated). The number of CpGs in each quadrant is given and the associated *P*-value is from a Fisher-exact test. **(b) **Example methylation (beta) profile of a CpG (cg00059225) undergoing age-associated hypermethylation and of one (cg07408456) undergoing hypomethylation. Red lines denote linear regression fits with associated *P*-values. **(c) **PCGT enrichment odds ratios (OR) for the top 500 age-hypermethylated (up) CpGs, the top 500 age-hypomethylated (dn) CpGs and the top 500 vCpGs. The green line denotes the null OR = 1, and 95% confidence intervals are shown. **(d) **Example methylation profile of an age-independent variable CpG (vCpG). **(e) **Scatterplot of all 24,039 CpG *t*-statistics (CIN2+ status; y-axis) against the corresponding estimated false discovery rate (FDR; x-axis). Red points indicate the top 500 vCpGs. The green line indicates FDR = 0.05. Click here for file

Additional file 10 **Comparison of mean methylation levels of the age-hypermethylated CpGs identified in ARTISTIC in set 1**. Set 1 consists of 30 normal LBC samples and 18 LBC samples exhibiting dysplasia (CIN2+). The *P*-value is from a Wilcoxon rank sum test. Click here for file

Additional file 11 **The 140 risk CpGs as identified using EVORA in the ARTISTIC cohort**. We provide the Illumina probe ID, Entrez ID, gene symbol, the ratio of age-adjusted variance in future CIN2+ cases relative to controls, the statistic of age-associated differential methylation change, and finally the t-statistic of differential methylation change between prospective CIN2+ and controls. Click here for file

Additional file 12 **Independent gene expression analysis of risk genes**. **(a) **Average relative mRNA expression levels of 86 risk genes that could be mapped to the expression arrays of the integrated mRNA data set, comparing levels across 42 normal cervical samples (green) and 74 cervical cancers (red). The *P*-value is from a one-sided Wilcoxon rank sum test. **(b) **Corresponding *t*-statistics of differential expression (y-axis) of the 86 genes against -log_10_(*P*-value). The number of genes passing *P *= 0.05 threshold and that are over-/underexpressed in cancer are given. The *P*-value is from a binomial test assuming (32 + 14 = 46 trials) and under the null that there is an equal chance of under- or overexpression. **(c) **Comparison of the observed binomial test *P*-value in (b) (vertical red line) to those binomial test *P*-values obtained from 1,000 Monte Carlo runs (green histogram), in which 86 genes were selected at random from the integrated expression set. The *P*-value shows the fraction of runs which more extreme *P*-values than the observed one. This Monte Carlo analysis therefore corrects for any bias in assuming that there is an equal null probability of under- or overexpression. Click here for file

Additional file 13 **DNA methylation and HPV status**. **(a) **Expected number of false positives (NFP, y-axis) is plotted against the number of positives (NP, x-axis) for CpGs associated with HPV status using surrogate variable analysis (SVA) and all 152 samples in the ARTISTIC cohort. **(b) **Boxplot comparing the EVORA risk scores of the 152 samples against HPV status. The *P*-value is from a Wilcoxon rank sum test. **(c) **As (a), using SVA with HPV status as the phenotype but now only using the 77 samples that remained disease-free. **(d) **As (a), using SVA with HPV status as the phenotype but now only using the 75 samples that developed a CIN2+. In (a, c, d), the green dashed line indicates the null-line of no association. We note that even in (d) the association is very marginal since the FDR for the top 100 CpGs is over 50%. Click here for file

Additional file 14 **Correlation of EVORA risk scores with CpG island methylation**. **(a) **Left panel: average beta methylation level over all CpGs mapping to CpG islands (excluding the 140 risk CpGs) on the y-axis versus the CIN2+ sample index. A partitioning around medoids algorithm (*pam *from package *cluster*) was used to cluster the samples into two clusters of relative high and low methylation, and the samples have been ordered and colored accordingly. Right panel: boxplot of the EVORA risk scores defined over the 140 risk CpGs in the same set of CIN2+ samples, grouped according to the clustering in (a). The *P*-value is from a Wilcoxon rank sum test. **(b) **Exactly as (a), but now for the cervical cancer samples of set 2. Click here for file

Additional file 15 **Consensus clustering heatmap of the 152 ARTISTIC samples over the top 1,000 most variable CpGs (vCpGs)**. Color codes in the heatmap: yellow, beta < 0.3; skyblue, 0.3 < beta < 0.7; blue, beta > 0.7. The bars above the heatmap indicate the consensus cluster (three clusters were optimal), HPV status (black = HPV-positive, grey = HPV-negative), prospective CIN2+ status (black = prospective CIN2+, grey = control) and EVORA risk score (green = risk score < 0.1, red = risk score > 0.1). Click here for file

Additional file 16 **Gene Set Enrichment Analysis using a one-tailed Fisher's exact test of the top 500 variable CpGs (vCpGs) against the Molecular Signatures Database (MSigDB, Broad Institute) **[25]. The columns label the gene list, the number of genes in the list, the number represented on the 27 K array, the corresponding fraction, the number that overlap with the top 500 vCpGs, the *P*-value of enrichment, the adjusted *P*-value, and the gene symbols for the enriched genes. Click here for file

Additional file 17 **Comparison of Methylight (PMR-value) based quantification of methylation (y-axis) with Infinium 27 K β-value (x-axis) for two of the identified risk genes (*SOX1 *and *WT1*) across the 48 LBC samples (set 1)**. The CpG on the 27 K array closest to the transcription start site and to the Methylight CpGs was used. The *P*-value is from a correlation test, testing the significance of the Spearman rank correlation. Red denotes the 18 CIN2+ samples, green denotes the 30 CIN2- samples. Click here for file

## References

[B1] Feinberg AP Irizarry RA Evolution in health and medicine Sackler colloquium: Stochastic epigenetic variation as a driving force of development, evolutionary adaptation, and disease. Proc Natl Acad Sci USA 2010 107 Suppl 1 1757 1764 20080672 10.1073/pnas.0906183107 PMC2868296

[B2] Feinberg AP Irizarry RA Fradin D Aryee MJ Murakami P Aspelund T Eiriksdottir G Harris TB Launer L Gudnason V Fallin MD Personalized epigenomic signatures that are stable over time and covary with body mass index. Sci Transl Med 2010 2 49ra67 10.1126/scitranslmed.3001262 PMC3137242 20844285

[B3] Hansen KD Timp W Bravo HC Sabunciyan S Langmead B McDonald OG Wen B Wu H Liu Y Diep D Briem E Zhang K Irizarry RA Feinberg AP Increased methylation variation in epigenetic domains across cancer types. Nat Genet 2011 43 768 775 10.1038/ng.865 21706001 PMC3145050

[B4] Bibikova M Fan JB Genome-wide DNA methylation profiling. Wiley Interdiscip Rev Syst Biol Med 2010 2 210 223 20836023 10.1002/wsbm.35

[B5] Kitchener HC Almonte M Gilham C Dowie R Stoykova B Sargent A Roberts C Desai M Peto J ARTISTIC: a randomised trial of human papillomavirus (HPV) testing in primary cervical screening. Health Technol Assess 2009 13 1 150, iii-iv 19891902 10.3310/hta13510

[B6] Kitchener HC Almonte M Thomson C Wheeler P Sargent A Stoykova B Gilham C Baysson H Roberts C Dowie R Desai M Mather J Bailey A Turner A Moss S Peto J HPV testing in combination with liquid-based cytology in primary cervical screening (ARTISTIC): a randomised controlled trial. Lancet Oncol 2009 10 672 682 10.1016/S1470-2045(09)70156-1 19540162

[B7] Teschendorff AE Menon U Gentry-Maharaj A Ramus SJ Weisenberger DJ Shen H Campan M Noushmehr H Bell CG Maxwell AP Savage DA Mueller-Holzner E Marth C Kocjan G Gayther SA Jones A Beck S Wagner W Laird PW Jacobs IJ Widschwendter M Age-dependent DNA methylation of genes that are suppressed in stem cells is a hallmark of cancer. Genome Res 2010 20 440 446 10.1101/gr.103606.109 20219944 PMC2847747

[B8] Edgar R Domrachev M Lash AE Gene Expression Omnibus: NCBI gene expression and hybridization array data repository. Nucleic Acids Res 2002 30 207 210 10.1093/nar/30.1.207 11752295 PMC99122

[B9] Scotto L Narayan G Nandula SV Arias-Pulido H Subramaniyam S Schneider A Kaufmann AM Wright JD Pothuri B Mansukhani M Murty VV Identification of copy number gain and overexpressed genes on chromosome arm 20q by an integrative genomic approach in cervical cancer: potential role in progression. Genes Chromosomes Cancer 2008 47 755 765 10.1002/gcc.20577 18506748

[B10] Zhai Y Kuick R Nan B Ota I Weiss SJ Trimble CL Fearon ER Cho KR Gene expression analysis of preinvasive and invasive cervical squamous cell carcinomas identifies HOXC10 as a key mediator of invasion. Cancer Res 2007 67 10163 10172 10.1158/0008-5472.CAN-07-2056 17974957

[B11] Pyeon D Newton MA Lambert PF den Boon JA Sengupta S Marsit CJ Woodworth CD Connor JP Haugen TH Smith EM Kelsey KT Turek LP Ahlquist P Fundamental differences in cell cycle deregulation in human papillomavirus-positive and human papillomavirus-negative head/neck and cervical cancers. Cancer Res 2007 67 4605 4619 10.1158/0008-5472.CAN-06-3619 17510386 PMC2858285

[B12] Teschendorff AE Miremadi A Pinder SE Ellis IO Caldas C An immune response gene expression module identifies a good prognosis subtype in estrogen receptor negative breast cancer. Genome Biol 2007 8 R157 10.1186/gb-2007-8-8-r157 17683518 PMC2374988

[B13] Teschendorff AE Gomez S Arenas A El-Ashry D Schmidt M Gehrmann M Caldas C Improved prognostic classification of breast cancer defined by antagonistic activation patterns of immune response pathway modules. BMC Cancer 2010 10 604 10.1186/1471-2407-10-604 21050467 PMC2991308

[B14] Teschendorff AE Naderi A Barbosa-Morais NL Caldas C PACK: Profile Analysis using Clustering and Kurtosis to find molecular classifiers in cancer. Bioinformatics 2006 22 2269 2275 10.1093/bioinformatics/btl174 16682424

[B15] Leek JT Storey JD A general framework for multiple testing dependence. Proc Natl Acad Sci USA 2008 105 18718 18723 10.1073/pnas.0808709105 19033188 PMC2586646

[B16] Storey JD Tibshirani R Statistical significance for genomewide studies. Proc Natl Acad Sci USA 2003 100 9440 9445 10.1073/pnas.1530509100 12883005 PMC170937

[B17] Du P Zhang X Huang CC Jafari N Kibbe WA Hou L Lin SM Comparison of Beta-value and M-value methods for quantifying methylation levels by microarray analysis. BMC Bioinformatics 2010 11 587 10.1186/1471-2105-11-587 21118553 PMC3012676

[B18] Laird PW Principles and challenges of genomewide DNA methylation analysis. Nat Rev Genet 2010 11 191 203 20125086 10.1038/nrg2732

[B19] Snedecor GW Cochran WG Statistical Methods 1989 Iowa State University Press

[B20] Tomlins SA Rhodes DR Perner S Dhanasekaran SM Mehra R Sun XW Varambally S Cao X Tchinda J Kuefer R Lee C Montie JE Shah RB Pienta KJ Rubin MA Chinnaiyan AM Recurrent fusion of TMPRSS2 and ETS transcription factor genes in prostate cancer. Science 2005 310 644 648 10.1126/science.1117679 16254181

[B21] Tian L Tibshirani R Adaptive index models for marker-based risk stratification. Biostatistics 2011 12 68 86 10.1093/biostatistics/kxq047 20663850 PMC3006126

[B22] R: A language and environment for statistical computing. R Foundation for Statistical Computing. http://www.r-project.org

[B23] Feinberg AP Ohlsson R Henikoff S The epigenetic progenitor origin of human cancer. Nat Rev Genet 2006 7 21 33 10.1038/nrg1748 16369569

[B24] Lee TI Jenner RG Boyer LA Guenther MG Levine SS Kumar RM Chevalier B Johnstone SE Cole MF Isono K Koseki H Fuchikami T Abe K Murray HL Zucker JP Yuan B Bell GW Herbolsheimer E Hannett NM Sun K Odom DT Otte AP Volkert TL Bartel DP Melton DA Gifford DK Jaenisch R Young RA Control of developmental regulators by Polycomb in human embryonic stem cells. Cell 2006 125 301 313 10.1016/j.cell.2006.02.043 16630818 PMC3773330

[B25] Subramanian A Tamayo P Mootha VK Mukherjee S Ebert BL Gillette MA Paulovich A Pomeroy SL Golub TR Lander ES Mesirov JP Gene set enrichment analysis: a knowledge-based approach for interpreting genome-wide expression profiles. Proc Natl Acad Sci USA 2005 102 15545 15550 10.1073/pnas.0506580102 16199517 PMC1239896

[B26] Walboomers JM Jacobs MV Manos MM Bosch FX Kummer JA Shah KV Snijders PJ Peto J Meijer CJ Munoz N Human papillomavirus is a necessary cause of invasive cervical cancer worldwide. J Pathol 1999 189 12 19 10.1002/(SICI)1096-9896(199909)189:1<12::AID-PATH431>3.0.CO;2-F 10451482

[B27] Kitchener HC Gilham C Sargent A Bailey A Albrow R Roberts C Desai M Mather J Turner A Moss S Peto J A comparison of HPV DNA testing and liquid based cytology over three rounds of primary cervical screening: extended follow up in the ARTISTIC trial. Eur J Cancer 2011 47 864 871 10.1016/j.ejca.2011.01.008 21334200

[B28] Sartor MA Dolinoy DC Jones TR Colacino JA Prince ME Carey TE Rozek LS Genome-wide methylation and expression differences in HPV(+) and HPV(-) squamous cell carcinoma cell lines are consistent with divergent mechanisms of carcinogenesis. Epigenetics 2011 6 777 787 10.4161/epi.6.6.16216 21613826 PMC3142368

[B29] Slebos RJ Yi Y Ely K Carter J Evjen A Zhang X Shyr Y Murphy BM Cmelak AJ Burkey BB Netterville JL Levy S Yarbrough WG Chung CH Gene expression differences associated with human papillomavirus status in head and neck squamous cell carcinoma. Clin Cancer Res 2006 12 701 709 10.1158/1078-0432.CCR-05-2017 16467079

[B30] Weisenberger DJ Siegmund KD Campan M Young J Long TI Faasse MA Kang GH Widschwendter M Weener D Buchanan D Koh H Simms L Barker M Leggett B Levine J Kim M French AJ Thibodeau SN Jass J Haile R Laird PW CpG island methylator phenotype underlies sporadic microsatellite instability and is tightly associated with BRAF mutation in colorectal cancer. Nat Genet 2006 38 787 793 10.1038/ng1834 16804544

[B31] Noushmehr H Weisenberger DJ Diefes K Phillips HS Pujara K Berman BP Pan F Pelloski CE Sulman EP Bhat KP Verhaak RG Hoadley KA Hayes DN Perou CM Schmidt HK Ding L Wilson RK Van Den Berg D Shen H Bengtsson H Neuvial P Cope LM Buckley J Herman JG Baylin SB Laird PW Aldape K Cancer Genome Atlas Research Network Identification of a CpG island methylator phenotype that defines a distinct subgroup of glioma. Cancer Cell 2010 17 510 522 10.1016/j.ccr.2010.03.017 20399149 PMC2872684

[B32] Fang F Turcan S Rimner A Kaufman A Giri D Morris LG Shen R Seshan V Mo Q Heguy A Baylin SB Ahuja N Viale A Massague J Norton L Vahdat LT Moynahan ME Chan TA Breast cancer methylomes establish an epigenomic foundation for metastasis. Sci Transl Med 2011 3 75ra25 10.1126/scitranslmed.3001875 PMC3146366 21430268

[B33] Sauvageau M Sauvageau G Polycomb group proteins: multi-faceted regulators of somatic stem cells and cancer. Cell Stem Cell 2010 7 299 313 10.1016/j.stem.2010.08.002 20804967 PMC4959883

[B34] Bracken AP Helin K Polycomb group proteins: navigators of lineage pathways led astray in cancer. Nat Rev Cancer 2009 9 773 784 10.1038/nrc2736 19851313

[B35] Johnstone SE Baylin SB Stress and the epigenetic landscape: a link to the pathobiology of human diseases?. Nat Rev Genet 2010 11 806 812 20921961 10.1038/nrg2881 PMC3148009

[B36] Cedar H Bergman Y Linking DNA methylation and histone modification: patterns and paradigms. Nat Rev Genet 2009 10 295 304 19308066 10.1038/nrg2540

[B37] Rodriguez-Paredes M Esteller M Cancer epigenetics reaches mainstream oncology. Nat Med 2011 17 330 339 21386836 10.1038/nm.2305

[B38] Wisman GB Nijhuis ER Hoque MO Reesink-Peters N Koning AJ Volders HH Buikema HJ Boezen HM Hollema H Schuuring E Sidransky D van der Zee AG Assessment of gene promoter hypermethylation for detection of cervical neoplasia. Int J Cancer 2006 119 1908 1914 10.1002/ijc.22060 16736496

[B39] Herman L Hubert P Herfs M Kustermans G Henrotin Y Bousarghin L Boniver J Delvenne P The L1 major capsid protein of HPV16 differentially modulates APC trafficking according to the vaccination or natural infection context. Eur J Immunol 2010 40 3075 3084 10.1002/eji.201040571 21061438

[B40] Kersemaekers AM van de Vijver MJ Kenter GG Fleuren GJ Genetic alterations during the progression of squamous cell carcinomas of the uterine cervix. Genes Chromosomes Cancer 1999 26 346 354 10.1002/(SICI)1098-2264(199912)26:4<346::AID-GCC9>3.0.CO;2-D 10534770

[B41] Widschwendter A Muller HM Fiegl H Ivarsson L Wiedemair A Muller-Holzner E Goebel G Marth C Widschwendter M DNA methylation in serum and tumors of cervical cancer patients. Clin Cancer Res 2004 10 565 571 10.1158/1078-0432.CCR-0825-03 14760078

[B42] Lin YW Chung MT Lai HC De Yan M Shih YL Chang CC Yu MH Methylation analysis of SFRP genes family in cervical adenocarcinoma. J Cancer Res Clin Oncol 2009 135 1665 1674 10.1007/s00432-009-0613-5 19513747 PMC11844787

[B43] Lai HC Lin YW Huang TH Yan P Huang RL Wang HC Liu J Chan MW Chu TY Sun CA Chang CC Yu MH Identification of novel DNA methylation markers in cervical cancer. Int J Cancer 2008 123 161 167 10.1002/ijc.23519 18398837

[B44] Missaoui N Hmissa S Trabelsi A Traore C Mokni M Dante R Frappart L Promoter hypermethylation of CDH13, DAPK1 and TWIST1 genes in precancerous and cancerous lesions of the uterine cervix. Pathol Res Pract 2011 207 37 42 10.1016/j.prp.2010.11.001 21129853

[B45] Lin CJ Lai HC Wang KH Hsiung CA Liu HW Ding DC Hsieh CY Chu TY Testing for methylated PCDH10 or WT1 is superior to the HPV test in detecting severe neoplasms (CIN3 or greater) in the triage of ASC-US smear results. Am J Obstet Gynecol 2011 204 21 e21 27 20833385 10.1016/j.ajog.2010.07.036

[B46] Maegawa S Hinkal G Kim HS Shen L Zhang L Zhang J Zhang N Liang S Donehower LA Issa JP Widespread and tissue specific age-related DNA methylation changes in mice. Genome Res 2010 20 332 340 10.1101/gr.096826.109 20107151 PMC2840983

[B47] Rakyan VK Down TA Maslau S Andrew T Yang TP Beyan H Whittaker P McCann OT Finer S Valdes AM Leslie RD Deloukas P Spector TD Human aging-associated DNA hypermethylation occurs preferentially at bivalent chromatin domains. Genome Res 2010 20 434 439 10.1101/gr.103101.109 20219945 PMC2847746

[B48] Teschendorff AE Menon U Gentry-Maharaj A Ramus SJ Gayther SA Apostolidou S Jones A Lechner M Beck S Jacobs IJ Widschwendter M An epigenetic signature in peripheral blood predicts active ovarian cancer. PLoS One 2009 4 e8274 10.1371/journal.pone.0008274 20019873 PMC2793425

[B49] Apostolidou S Hadwin R Burnell M Jones A Baff D Pyndiah N Mould T Jacobs IJ Beddows S Kocjan G Widschwendter M DNA methylation analysis in liquid-based cytology for cervical cancer screening. Int J Cancer 2009 125 2995 3002 10.1002/ijc.24745 19609949

[B50] Beck S Taking the measure of the methylome. Nat Biotechnol 2010 28 1026 1028 10.1038/nbt1010-1026 20944589

[B51] Sandoval J Heyn HA Moran S Serra-Musach J Pujana MA Bibikova M Esteller M Validation of a DNA methylation microarray for 450,000 CpG sites in the human genome. Epigenetics 2011 6 692 702 10.4161/epi.6.6.16196 21593595

