# Supplementary material for: Epigenetic variability in cells of normal cytology is associated with the risk of future morphological transformation
Source: Genome Med. 2012 Mar 27;4(3):24. doi: 10.1186/gm323 (PMC3446274; doi:10.1186/gm323)
Supplement: Additional file 1 — Supplementary information with further details of Materials and methods. [file gm323-S1.DOC]

**SUPPLEMENTARY INFORMATION**

**for manuscript**

**Epigenetic variability in cells of normal cytology is associated with the risk of future morphological transformation**

Andrew E Teschendorff, Allison Jones, Heidi Fiegl, Alexandra Sargent, Joanna Zhuang, Henry C Kitchener & Martin Widschwendter

### Study Population

#### The ARTISTIC trial

The liquid based cytology (LBC) samples we analyzed were collected from women as part of the ARTISTIC (A Randomised Trial In Screening To Improve Cytology) trial . This trial is registered with the International Standard Randomised Controlled Trial Number ISRCTN25417821. Within the ARTISTIC trial, women, aged 20 to 64 years, who were undergoing routine screening as part of the English National Health Service Cervical Screening Programme in Greater Manchester were randomly assigned in a ratio of 3:1 to either combined LBC and HPV testing in which the results were revealed and acted on, or to combined LBC and HPV testing where the HPV result was concealed from the patient and investigator. There were a total of 24,510 eligible women at entry. In the first round of screening 453 women had CIN2+. In the second round of screening 75 women (who were screen negative in the first round and who had a sample stored from the first round) had developed CIN2+ (44 were HPV positive and 31 were HPV negative in the first round). 77 women who had not developed any cytological changes were matched (age and HPV status in round 1) to the cases. The cytologically normal samples from round 1 from these 152 women were used for DNA methylation analysis.

#### Cytology and HPV scoring

Slides were prepared from liquid based cytology (LBC) samples on a ThinPrep T3000 processor (Hologic; Crawley, UK). Cytology was reported using the classification of the British Society of Cervical Cytology. Liquid residues of LBC samples were tested for HPV using the Digene Hybrid Capture 2 (HC2, Qiagen; Crawley, UK) test. This nucleic acid microplate chemiluminescent detection assay detects 13 high-risk HPV types (16, 18, 31, 33, 35, 39, 45, 51, 52, 56, 58, 59, and 68). Results were read and calculated on the Digene Microplate Luminometer 2000 (DML 2000; Qiagen, Crawley, UK) using the HC2 software at the recommended relative light units to control ratio of greater than or equal to 1.0. HPV results were reported independently of both cytology and histopathology. Histology of cytology/HPV-triggered loop excision samples was classified as cervical intraepithelial lesion grade 2 or worse (CIN2+).

#### DNA methylation nested case control study

Residual LBC specimens were frozen and stored at 20°C. A total of 152 samples in a prospective nested case control study within ARTISTIC were selected for DNA methylation analysis. Cases were 75 women who had normal cytology in screening round 1 but demonstrated CIN2+ after three years in round 2. Controls were 77 women who had normal cytology at entry and in the second screening round. Cases and controls were matched for age (Wilcox rank sum test *P*=0.95) and HPV status: 92 were HPV positive (44 cases / 48 controls) and 60 were HPV negative (31 cases / 29 controls) at entry (Fisher test *P*=0.74).

This study- performing DNA methylation profiling in LBC samples - has been approved by the ethical committee (National Research Ethics Service Reference Number: 10/H1107/15.

#### Other DNA methylation data sets

In addition to the nested case control prospective study within ARTISTIC, we used two additional DNA methylation data sets:

Set1: A total of 30 LBC samples (HPV negatives and positives) with normal cytology and 18 LBC samples (all HPV positive) with CIN2+, as described in .

Set2: A total of 63 cervical tissue samples: 48 cervical cancers, 15 normals. The normal cervical tissue samples were from women (mean age 55.4 years) who underwent a hysterectomy for uterine fibroids. The 48 cervical cancer specimens were from women (mean age 56.8 years) who were treated at the Innsbruck Medical University between 1990 and 2006. Of the cancers, 26 and 22 were stage 1 and stage 2/3/4, respectively, 37 were squamous cell cancers, 10 were adenocarcinomas and 1 was adenosquamous. Among the cancers, 7, 28 and 11 were grade 1, 2 and 3 respectively. All specimens were obtained with informed consent and approval from the ethics committee UN4044-290/4.9 .

**DNA extraction and Methylation Assay**

DNA from LBC samples was isolated from the described samples using the Qiagen DNeasy Blood and Tissue Kit (Qiagen Ltd, UK, 69506). DNA was quantified via spectrophotometry (Nanodrop, Thermo Scientific Ltd UK) and 600ng were bisulphite converted using the Zymo methylation Kits (Zymo Research Inc, USA, D5004/8) and genome wide methylation analysis using the Illumina Infinium Methylation27K beadchip (Illumina Inc USA, WG-311-1201) was performed. The beadchip interrogates the methylation status of over 27000 CpG sites throughout the human genome, covering the promoters of over 14000 genes . Approximately 2 CpG sites are therefore represented per gene promoter. The assay involves capture of sample DNA to methylated and unmethylated versions of allele specific primers coated on glass beads. Thus, for each CpG site, two intensity values are recorded, one for each of the methylated (*M*) and unmethylated (*U*) versions of the allele. Fraction of cells with the CpG site methylated is quantified in terms of the ratio (the β-value) of methylated intensity to total intensity as β=max(*M*,0)/(max(*U*,0)+max(*M*,0)+100), which represents a continuous value between 0 (unmethylated) and 1 (methylated).

**Data availability**

All new data in this manuscript has been deposited in the Gene Expression Omnibus (GEO) ([*www.ncbi.nlm.nih.gov/geo/*](http://www.ncbi.nlm.nih.gov/geo/)) data repository under the GEO accession number GSE30760.

**MethyLight assay**

Quantitative PCR analysis using MethyLight was performed in liquid based cytology samples from Set 1 (18 CIN2+ and 30 normals) as previously described . MethyLight primers and probes were designed specifically for bisulfite-converted DNA for each locus; a methylated set for the gene of interest and a reference gene (COL2A1) to normalize for input DNA. Specificity of the reactions for methylated DNA was confirmed separately using SssI-treated human white blood cell DNA (heavily methylated). The percentage of fully methylated molecules at a specific locus was calculated by dividing the GENE:COL2A1 ratio of a sample by the GENE:COL2A1 ratio of the SssI-treated human white blood cell DNA and multiplied by 100. The abbreviation PMR (percentage of methylated reference) indicates this measurement, which is commonly used to analyze quantitative MethyLight data. The analysis was performed blinded and samples (cases/controls) were randomly allocated into the training, and the validation set were mixed for bisulfite treatment and for real-time PCR. The concentration of bisulfite modified DNA (assessed by the level of the reference gene COL2A1) was the same between cases and controls.

Specific information for the genes analyzed:

SOX1: covering eight CpGs in a region within a CpG island which is -383 to -309 to TSS.

Forward Primer: AAC CCG ACC CGA AAT ATA CTA TCT

Reverse Primer: GAG GAT CGA GCG TAG GAG GAA

Probe: CGA CCG CCG CTA CGC GCT ACT T

WT1: covering six CpGs in a region within a CpG island which is -214 to -108 to TSS.

Forward Primer: CAA ACA ACG CGC GCT ACT C

Reverse Primer: GCG CGT GTT GGG TTG AAG

Probe: AAA ACG CCC TCG CAA CTA

### Definition of polycomb group target (PCGT) genes:

We use the list of PCGT genes defined in . Specifically, those genes in human embryonic stem cells with occupancy of either SUZ12, EED or H3K27me3. Under this definition there were 2,705 CpGs mapping to PCGTs (and passing quality control).

### Statistical Methods

#### Quality Control and Inter-Array Normalisation

The raw DNA methylation data was subject to a similar quality control procedure as used in our previous publication . This procedure was designed to detect and remove samples with poor CpG coverage (i.e samples with many probes which were not detected above background, as estimated from negative controls). To evaluate beadchip effects and variations in bisulfite conversion efficiency (BSCE) and other experimental factors, we used a singular value decomposition (SVD) to decompose the row(CpG)-centred beta valued data matrix into components, which were then correlated to both phenotypes of interest and experimental factors. To estimate the number of components carrying significant variance, we first mean centered the beta matrix by substracting the mean methylation level from each CpG (this was done for each CpG separately). This step removes the trivial variation due to CpGs having different mean methylation levels and allows proper estimation of the number of significant components. This estimation was then done using the Random Matrix Theory (RMT) framework . The analytical RMT estimate was validated by scrambling up the data matrix (a distinct permutation of samples for each CpG) and showing that it estimated zero significant components.

The SVD analysis revealed many significant components correlating with beadchip and BSCE. We therefore adjusted the data for these two factors and validated the adjustment by repeating the SVD on the adjusted data, which showed that significant components no longer correlated with either beadchip or BSCE.

#### Supervised Analyses

To identify CpGs associated with age (aCpGs) we used Surrogate Variable Analysis (SVA) . Briefly, for each CpG we used a multivariate linear regression framework with age as the response and the adjusted CpG methylation profile plus surrogate variables as predictors. Subsequently, P-values from these regressions were adjusted for multiple testing using the FDR estimation implemented in the q-value package .

To identify age-independent variable CpGs (vCpGs) we further adjusted the data for age, and subsequently estimated the variances for each CpG. Because of the heteroscedasticity of β-values , we estimated variance in two different ways from: (i) β-values and (ii) *R*-values (defined as *R=M/U*). However, the overlap between the top 500 vCpGs obtained with either scale was very strong (270 CpGs overlapped, *P*<1*e*-50), and the Pearson correlation between the two variance measures was 0.77 over all CpGs. Subsequent results on the top 500 vCpGs did not depend appreciably on which scale was used to estimate variance.

To identify differentially variable CpGs (DVCs) between prospective CIN2+ cases and normals we used Bartlett’s test . Specifically, in comparing the variance of prospective CIN2+ cases to that of controls we used the $\chi^{2}$-statistic (1 degree of freedom)

(1)

where (number of prospective cases, number of controls), and . This was used over the non-parametric equivalent (Levene test), because Bartlett’s test is more sensitive to outliers (in our case outlier methylation profiles are those of biological interest).

#### EVORA: Epigenetic ­Variable Outliers for Risk prediction Analysis

EVORA is similar to an adaptive index algorithm and is presented in a separate paper . However, for convenience we describe the algorithm again below. EVORA is based on the following three hypotheses:

- Relevant DNA methylation features (i.e CpGs capable of predicting disease phenotypes) may be identified more accurately by comparing the variance in methylation between phenotypes, as proposed in . We refer to these CpGs as “risk CpGs”.
- Risk CpGs exhibit outlier methylation profiles and can be identified more accurately using differential variability. This is motivated by the hypothesis that much of the epigenetic variation is stochastic .
- The cancer risk score of an individual sample is proportional to the number of risk CpGs that constitute significant methylation outliers (specifically hypermethylation) in that given sample. We call these outliers, “methylation hits”. This proportionality assumption is reasonable as it reflects the degree of deviation from normal baseline methylation levels in the healthy phenotype.

To translate these model assumptions into a prediction tool, we need to be able to (i) identify risk CpGs and samples that constitute outliers for these candidate risk CpGs, and (ii) a statistical method for assigning risk to each sample, and preferably one which is robust and independent of the scale used. Scale independence is important to guarantee that classification thresholds are generalizable to independent data sets. To address problem (i) we propose to transform the DNAm data matrix so that outliers can be identified in an objective manner independently of the scale used.

To address problem (i), and given a set of cytologically normal DNAm samples, we propose to first transform the DNAm data matrix so that outliers can be identified in an objective manner independently of the scale used. To achieve this, we propose to use the COPA (Cancer Outlier Profile Analysis) transformation, which was first used in the context of gene expression data to identify candidate gene fusions with outlier gene expression . Specifically, for each CpG with methylation profile $\vec{\beta}_{cpg}$ we transform it to a COPA-profile

(2)

where *mad* denotes the median absolute deviation. In other words, we subtract out the median of a profile, then find the median of the non-zero absolute deviations, and finally divide the median centred profile by this number. Thus, the COPA-transformed values are independent of the scale used and for a given threshold one may now define samples that constitute outliers for that specific CpG profile.

To address problem (ii) we propose the EVORA algorithm, which consists of the following steps:

1. Perform the COPA transformation on the whole DNAm data matrix. Phenotype information is obviously not used in this step.
2. Start an internal 10-fold cross-validation. At each fold, samples are split into a training and test set ensuring that training and test set contain equal relative proportions of the two phenotypes (i.e normal and prospective CIN2+), and that each sample is used only once in each test set.
3. Using the original beta-valued data matrix, identify and rank candidate risk CpGs using Bartletts test in the training set (i.e identify CpGs more variable in the CIN2+ phenotype-we call these CpGs “hyperV DVCs”). Perform FDR (false discovery rate) analysis to determine if there are sufficient CpGs that pass an FDR=0.05 threshold. At this point the ranking can also take into consideration other criteria such as association with age as evaluated in the same training set.
4. For each candidate risk CpG and for a range of choices of COPA thresholds, transform the COPA methylation profile to a binarised EVORA profile, in which each sample is scored as 1 (if the COPA value for that sample is larger than the current threshold) or 0 (if the COPA value is less than the threshold).
5. For each sample in the test set calculate the fraction of risk CpGs which are outliers (i.e number of 1s). This fraction is the risk index of that sample and is dependent on the COPA threshold and on the number of top ranked risk CpGs included.
6. Repeat steps 3-5 for each fold. Thus, each sample acts as a test sample once and is assigned a risk score.
7. Compute the AUC of the resulting risk scores at each COPA threshold and for different numbers of top ranked risk CpGs (typically starting at 50 and ending at 1000).
8. Find the COPA threshold and number of risk CpGs that optimizes the AUC over the internal cross-validation.
9. Having identified the optimal parameters, the risk score of an independent sample is obtained as the fraction of selected risk CpGs that have this sample as an outlier according to the optimal COPA threshold.

We make several notes about this procedure:

(i) Having identified the optimal number of risk CpGs, *n**, to be included, the exact composition of this final list of risk CpGs may be constructed by considering the union of all sets of risk CpGs obtained in the internal crossvalidation. Specifically, for the union set of such risk CpGs we count how often a given CpG is present in each run and rank CpGs according to how often they are chosen. We declare the final risk CpG set as the top *n** of this ranked list.

(ii) Since the risk score is just the percentage of methylation hits in a given sample, different samples may be assigned the same risk score. To discriminate samples with tied risk scores we computed a mean methylation score over the risk CpGs using the original beta values. The mean methylation value of each of these tied samples was then renormalised to lie in the open interval (0,1) using the transformation , where ε is a small offset term to ensure that the scores lie in the open interval (i.e values 1 and 0 are to be excluded since otherwise this would induce ties with samples which have neighboring risk scores). This whole procedure therefore ranks samples first according to percentage of methylation hits and resolves ties using a mean methylation score.

(iii) The range of COPA thresholds to consider and to optimise over is determined by a maximum value which should correspond to the threshold for calling a CpG methylated. On the Infinium platform this maps roughly to around 0.2 or 0.25 and can be estimated using a mixture of beta-distributions. A value of 0.2 or 0.25 typically translates to a COPA threshold of around 10. Thus, in practice we consider integer valued COPA thresholds between 1 and 10.

(iv) It is important to stress that the EVORA risk score is an outlier risk score, i.e it is derived from a scale (the COPA-scale) that allows improved identification of outliers. This also means that, potentially, the optimal COPA threshold is trained on a specific type of outlier profile. Mathematically, outlier profiles are bi-modal (or multi-modal) and these can be of two types: type-I profiles where the outlier group is made up of a relatively small number of samples, or type-II where the outlier group has substantially more samples and is more similar to the size of the normal group. Thus, if the training and test data sets differ significantly in terms of the type of outlier profiles they exhibit, this could compromise the performance of EVORA. In such a scenario, the COPA threshold can be transformed back into the beta-value scale, and the score in the independent sample computed using this threshold on the original beta values.

In the context of the ARTISTIC sample set, risk CpGs exhibited outlier profiles of type-I. However, the majority of risk CpGs had outlier methylation profiles of type-II in the cervical cancer/normal set. In other words, while risk CpGs were heterogeneously methylated in the prospective CIN2+ cases, the majority of these were homogeneously methylated in cervical cancer. Mathematically, this means that the EVORA risk scores are significantly reduced in the cervical cancer/normal set owing to a much larger median absolute deviation (*mad*) value, despite the fact that many more risk CpGs become methylated in any given cervical cancer. Therefore, most risk CpGs lose their type-I outlier structure in cervical cancer, where they become more homogeneously methylated (type-II profile). Thus, to allow meaningful computation of EVORA scores in cancer cells requires adapting the algorithm to the β methylation scale. Specifically, one only needs to estimate the threshold on the β-value scale that corresponds to the given optimal COPA threshold. For an optimal COPA threshold of 5 we estimated a corresponding optimal beta threshold of β~0.2, hence in the cervical cancer/normal set (Set2) we estimated the proportion of methylation hits (i.e the score) in a given sample as the number of risk CpGs with β methylation values larger than 0.2. Importantly and independently of this, the β value threshold of 0.2 is a safe and natural threshold to use for calling CpGs methylated (β>0.2), as we verified by explicitly fitting a mixture model of beta distributions to the sample methylation profiles.

#### mRNA expression analysis

We downloaded the intra-array normalized expression data of three separate publications from the Gene Expression Omnibus ([*www.ncbi.nlm.nih.gov/*geo](http://www.ncbi.nlm.nih.gov/geo)) with accession numbers GSE9750 , GSE7803 , GSE6791 . These data were subject to further quantile normalisation to correct for inter-array variations. We only selected samples which were normal cervical tissue or cervical cancer to allow for a proper integrative meta-analysis. Probes mapping to the same Entrez ID were averaged. We mapped the set of risk genes (i.e the unique genes associated with our 140 risk CpGs) to each of the three expression arrays. A total of 86 genes were mapped to all three expression arrays. For each of these genes, and in each expression study, we normalised the expression profile to zero mean and unit variance. The resulting three expression profiles were then merged to yield an integrated mRNA profile across a total of 42 normal cervical samples and 74 cervical cancers, separately for each of the 86 genes. We then computed t-statistics and P-values of association between mRNA level and cancer/normal status for each gene. P-values less than 0.05 were declared significant and the number of over and under expressed genes counted. Any skew towards the expected underexpression was evaluated using a binomial test. The significance of this skew was evaluated using a Monte-Carlo analysis, in which a randomly selected set of 86 genes was used to yield a null P-value. A total of 1000 Monte-Carlo runs yielded a null P-value distribution and the fraction of runs with a P-value more extreme than the observed one, yielded a global significance P-value. In a separate analysis, we also compared the average mRNA levels over the 86 genes between normal and cancer tissue and the difference was evaluated using the Wilcoxon-test.

**References:**

1. Kitchener, H.C., et al., *ARTISTIC: a randomised trial of human papillomavirus (HPV) testing in primary cervical screening.* Health Technol Assess, 2009. **13**(51): p. 1-150, iii-iv.

2. Kitchener, H.C., et al., *HPV testing in combination with liquid-based cytology in primary cervical screening (ARTISTIC): a randomised controlled trial.* Lancet Oncol, 2009. **10**(7): p. 672-82.

3. Teschendorff, A.E., et al., *Age-dependent DNA methylation of genes that are suppressed in stem cells is a hallmark of cancer.* Genome Res, 2010. **20**(4): p. 440-6.

4. Bibikova, M. and J.B. Fan, *Genome-wide DNA methylation profiling.* Wiley Interdiscip Rev Syst Biol Med, 2010. **2**(2): p. 210-23.

5. Apostolidou, S., et al., *DNA methylation analysis in liquid-based cytology for cervical cancer screening.* Int J Cancer, 2009. **125**(12): p. 2995-3002.

6. Lee, T.I., et al., *Control of developmental regulators by Polycomb in human embryonic stem cells.* Cell, 2006. **125**(2): p. 301-13.

7. Plerou, V., et al., *Random matrix approach to cross correlations in financial data.* Phys Rev E Stat Nonlin Soft Matter Phys, 2002. **65**(6 Pt 2): p. 066126.

8. Leek, J.T. and J.D. Storey, *A general framework for multiple testing dependence.* Proc Natl Acad Sci U S A, 2008. **105**(48): p. 18718-23.

9. Storey, J.D. and R. Tibshirani, *Statistical significance for genomewide studies.* Proc Natl Acad Sci U S A, 2003. **100**(16): p. 9440-5.

10. Du, P., et al., *Comparison of Beta-value and M-value methods for quantifying methylation levels by microarray analysis.* BMC Bioinformatics, 2010. **11**: p. 587.

11. Snedecor, G.W. and W.G. Cochran, *Statistical Methods*1989.

12. Tian, L. and R. Tibshirani, *Adaptive index models for marker-based risk stratification.* Biostatistics, 2011. **12**(1): p. 68-86.

13. Teschendorff, A. and M. Widschwendter, *Differential variability improves the identification of cancer risk and early detection markers in DNA methylation studies.* Submitted.

14. Feinberg, A.P. and R.A. Irizarry, *Evolution in health and medicine Sackler colloquium: Stochastic epigenetic variation as a driving force of development, evolutionary adaptation, and disease.* Proc Natl Acad Sci U S A, 2010. **107 Suppl 1**: p. 1757-64.

15. Hansen, K.D., et al., *Increased methylation variation in epigenetic domains across cancer types.* Nat Genet, 2011. **43**(8): p. 768-75.

16. Tomlins, S.A., et al., *Recurrent fusion of TMPRSS2 and ETS transcription factor genes in prostate cancer.* Science, 2005. **310**(5748): p. 644-8.

17. Scotto, L., et al., *Identification of copy number gain and overexpressed genes on chromosome arm 20q by an integrative genomic approach in cervical cancer: potential role in progression.* Genes Chromosomes Cancer, 2008. **47**(9): p. 755-65.

18. Zhai, Y., et al., *Gene expression analysis of preinvasive and invasive cervical squamous cell carcinomas identifies HOXC10 as a key mediator of invasion.* Cancer Res, 2007. **67**(21): p. 10163-72.

19. Pyeon, D., et al., *Fundamental differences in cell cycle deregulation in human papillomavirus-positive and human papillomavirus-negative head/neck and cervical cancers.* Cancer Res, 2007. **67**(10): p. 4605-19.
