# Supplementary figures and images for: Epigenetic variability in cells of normal cytology is associated with the risk of future morphological transformation
Source: Genome Med. 2012 Mar 27;4(3):24. doi: 10.1186/gm323 (PMC3446274; doi:10.1186/gm323)

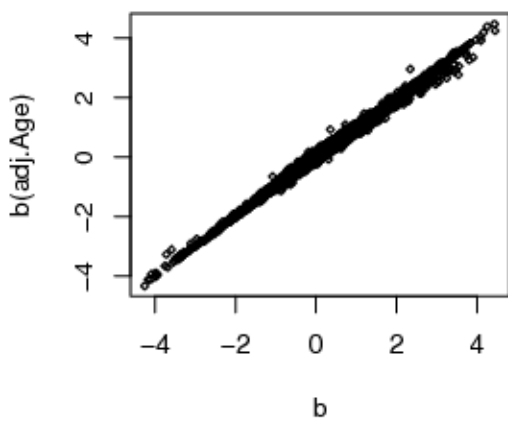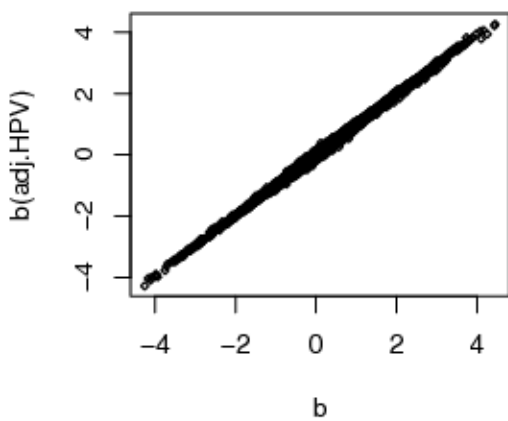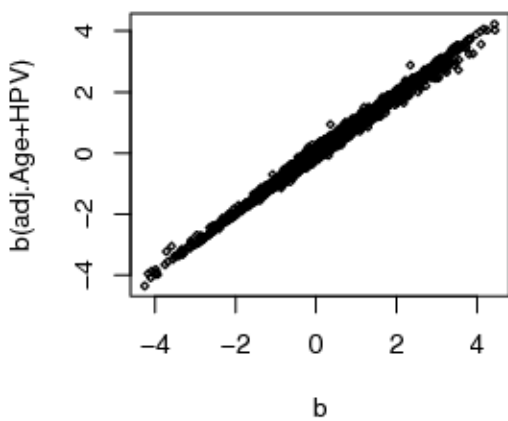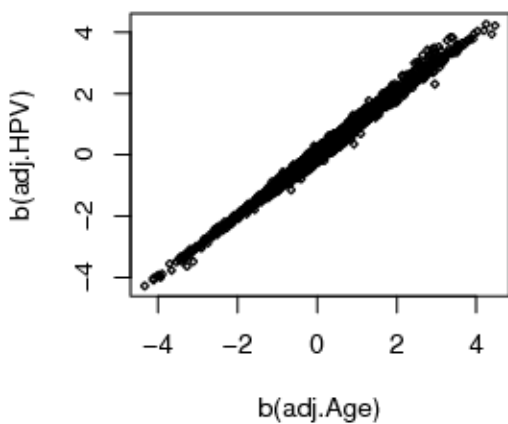

Supplement: Additional file 3 — Scatterplots of the Bartlett test b-statistics. Scatterplots of the Bartlett test b-statistics (that is, log2(ratio of variances of prospective CIN2+ to normal)) obtained without adjustment of age or HPV status (b), against the corresponding ones obtained after adjustment for age (b(adj.Age)), after adjustment for HPV status (b(adj.HPV)), and after adjustment for both age and HPV status(b(Adj.Age+HPV)). [file gm323-S3.PDF]

**cg02008154**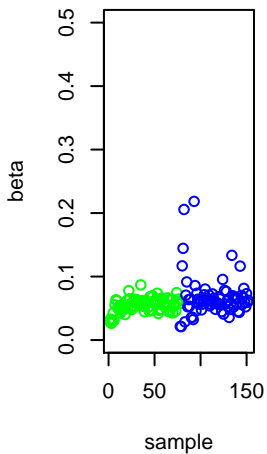**cg25044651**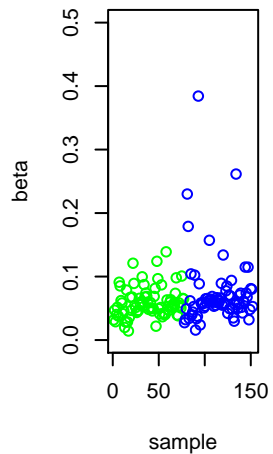**cg00399483**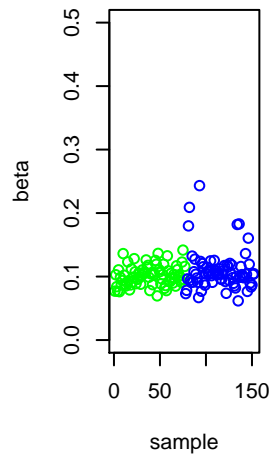**cg23710218**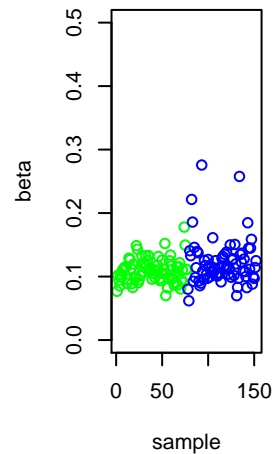**cg23316360**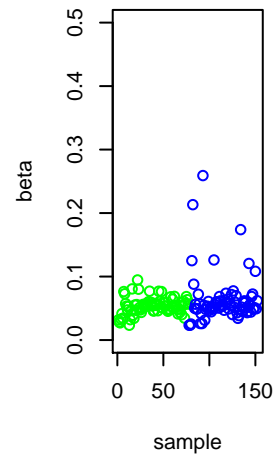**cg18236477**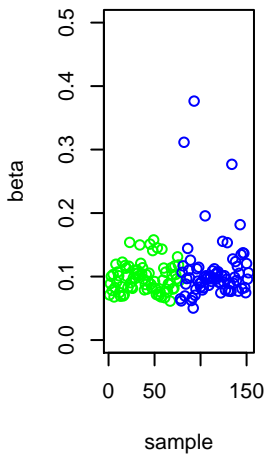**cg13870866**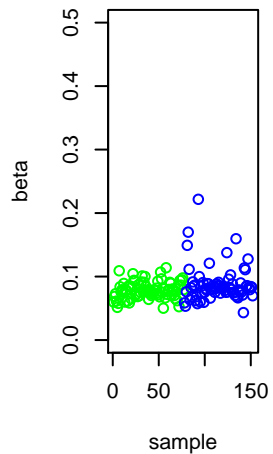**cg21296230**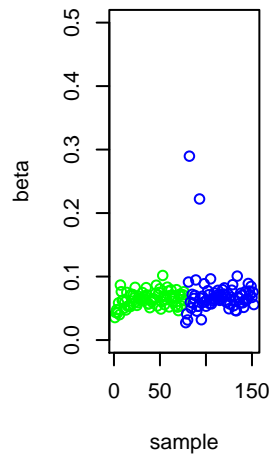**cg12457773**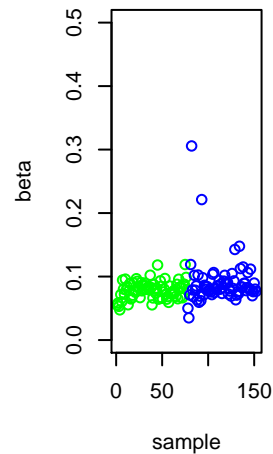**cg10331779**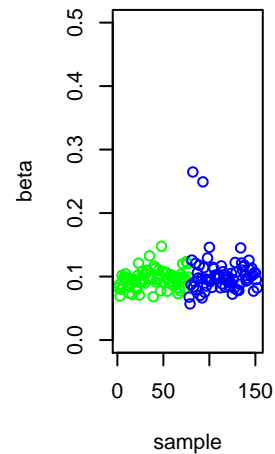

Supplement: Additional file 4 — Methylation profiles of 10 of the top hypervariable DVCs in the ARTISTIC cohort (all 10 shown are also among the 140 risk CpGs). Green denotes normal samples and blue denotes prospective CIN2+ cases. [file gm323-S4.PDF]

PCGT enrichment OR and relative OR (ROR)

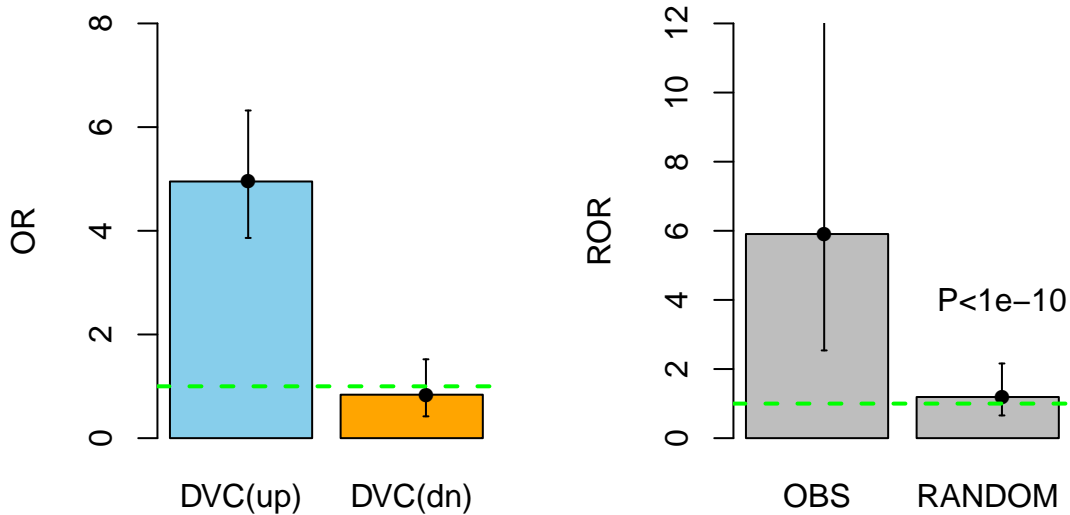

Supplement: Additional file 5 — PCGT enrichment odds ratio (OR) among the top 500 DVCs and the corresponding relative odds ratio (ROR) of PCGT enrichment (ROR = OR(more variable in future CIN2+)/OR(less variable in future CIN2+)). Also shown is the expected ROR for the null case where the top 500 CpGs were selected after a random permutation of sample labels. The P-value reflects the significance of the difference between the observed and null ROR. [file gm323-S5.PDF]

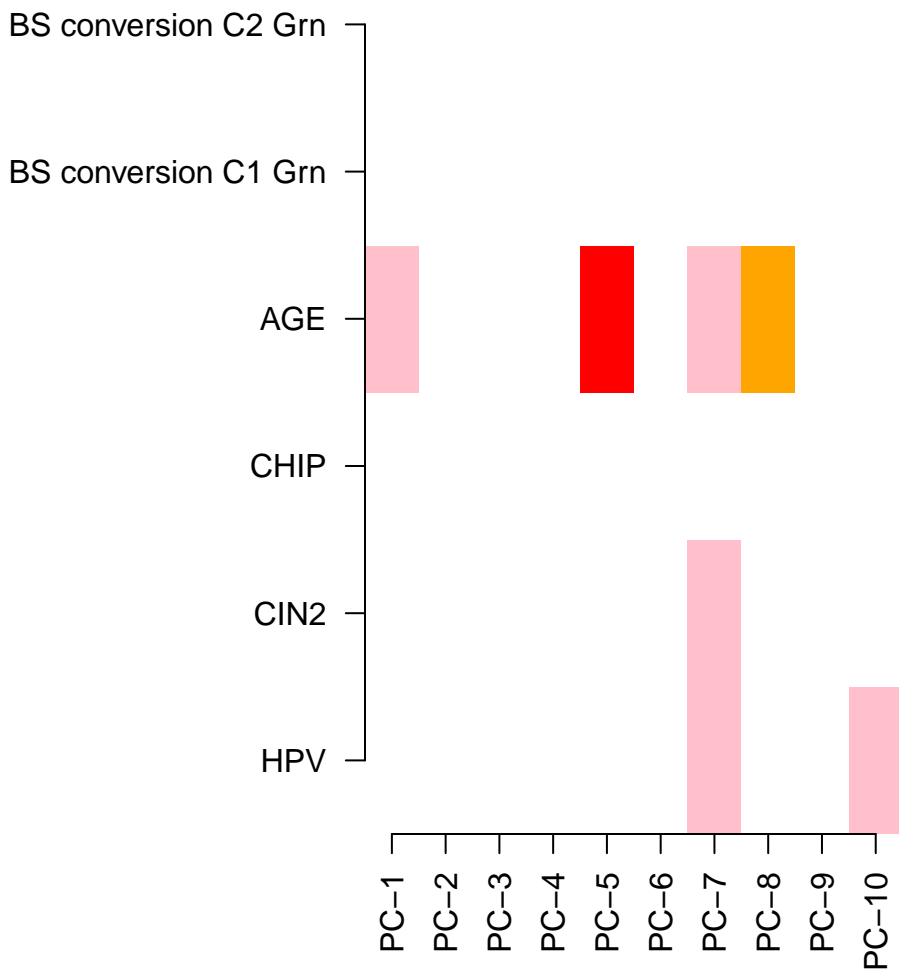

Supplement: Additional file 8 — Heatmap of P-values of association between the singular vectors of a singular value decomposition on the inter-array normalized adjusted data, with experimental (Bisulfite conversion efficiency controls (BSCE) 1 and 2, beadchip) and phenotypic factors (CIN2+ status, HPV status and age). P-values were estimated using t-tests (CIN2+ and HPV status), linear regression (age and BSCE BSCE: bi-sulfite conversion efficiency) and ANOVA (beadchip). Color codes: P < 1e-10 (dark red), P < 1e-5 (red), P < 0.001 (orange), P < 0.05 (pink), P > 0.05 (white). [file gm323-S8.PDF]

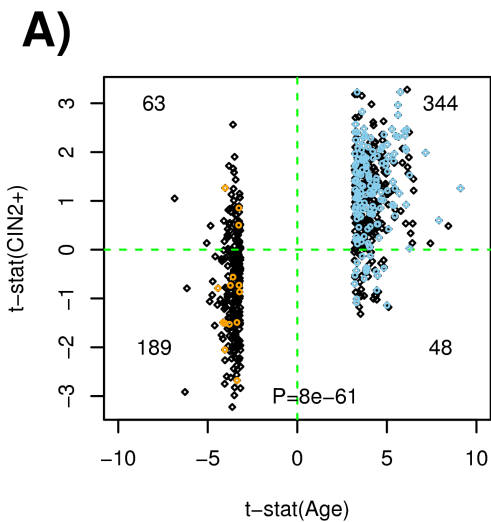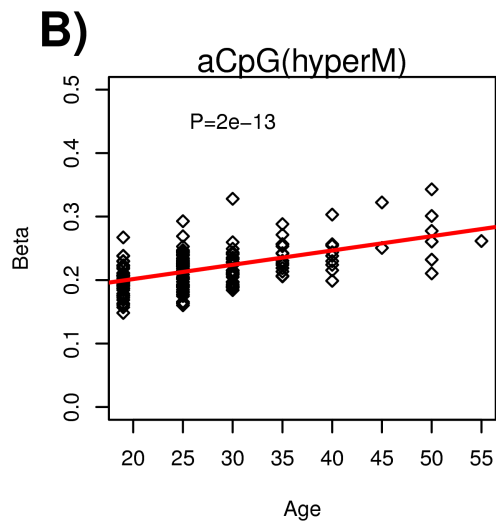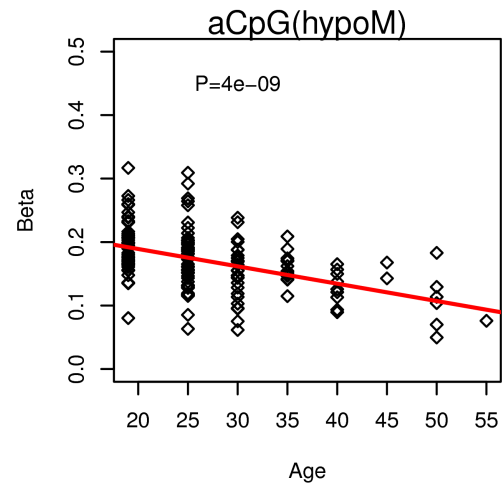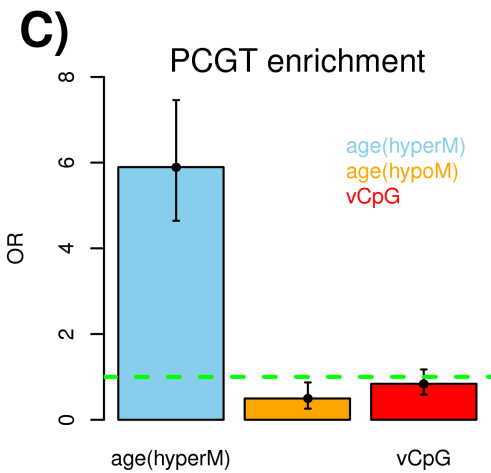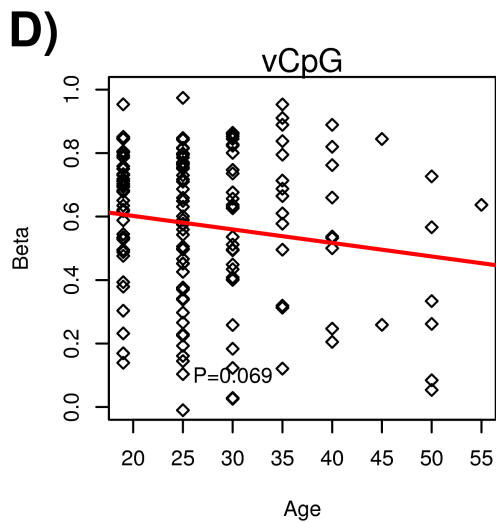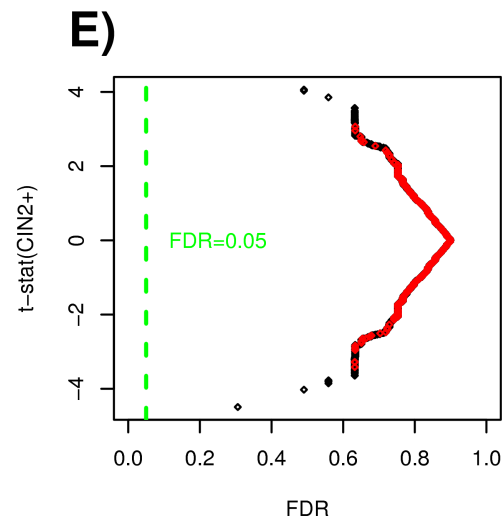

Supplement: Additional file 9 — Age-associated CpGs and variable CpGs and their relation to CIN2+ status. (a) Scatterplot of t-statistics of the 644 age-associated CpGs (FDR < 0.05). Their t-statistics relative to CIN2+ status (y-axis) are plotted against their age associated t-statistics (x-axis). Colored CpGs denote the 175 age-PCGT CpGs (skyblue = age-hypermethylated; orange = age-hypomethylated). The number of CpGs in each quadrant is given and the associated P-value is from a Fisher-exact test. (b) Example methylation (beta) profile of a CpG (cg00059225) undergoing age-associated hypermethylation and of one (cg07408456) undergoing hypomethylation. Red lines denote linear regression fits with associated P-values. (c) PCGT enrichment odds ratios (OR) for the top 500 age-hypermethylated (up) CpGs, the top 500 age-hypomethylated (dn) CpGs and the top 500 vCpGs. The green line denotes the null OR = 1, and 95% confidence intervals are shown. (d) Example methylation profile of an age-independent variable CpG (vCpG). (e) Scatterplot of all 24,039 CpG t-statistics (CIN2+ status; y-axis) against the corresponding estimated false discovery rate (FDR; x-axis). Red points indicate the top 500 vCpGs. The green line indicates FDR = 0.05. [file gm323-S9.PDF]

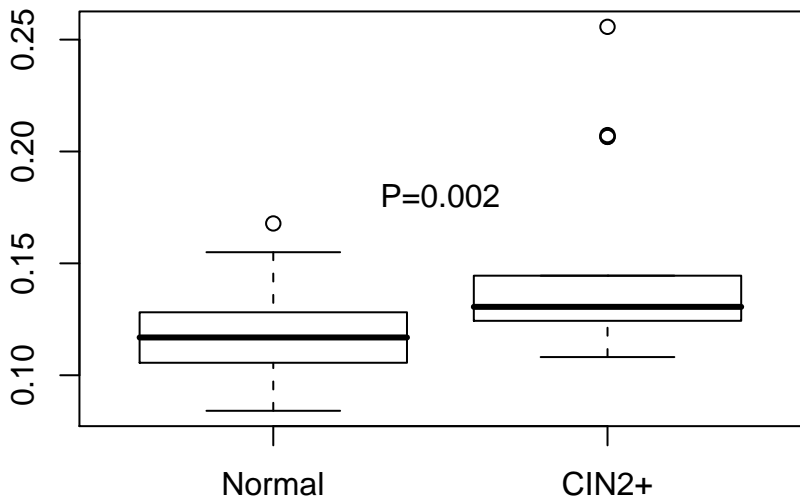

Supplement: Additional file 10 — Comparison of mean methylation levels of the age-hypermethylated CpGs identified in ARTISTIC in set 1. Set 1 consists of 30 normal LBC samples and 18 LBC samples exhibiting dysplasia (CIN2+). The P-value is from a Wilcoxon rank sum test. [file gm323-S10.PDF]

**A)**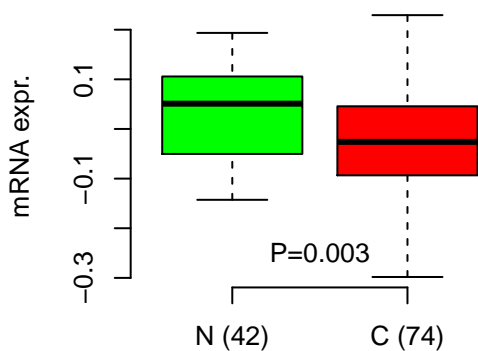**B)**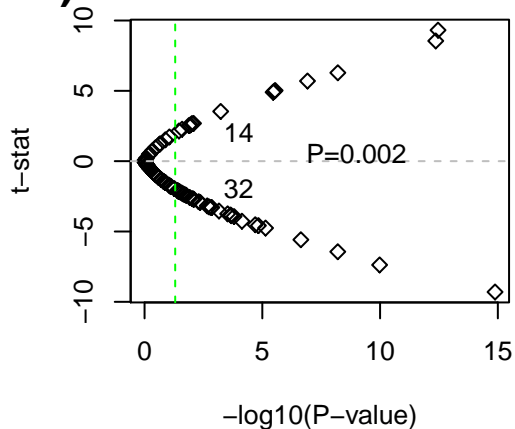**C)**

### Monte Carlo Analysis

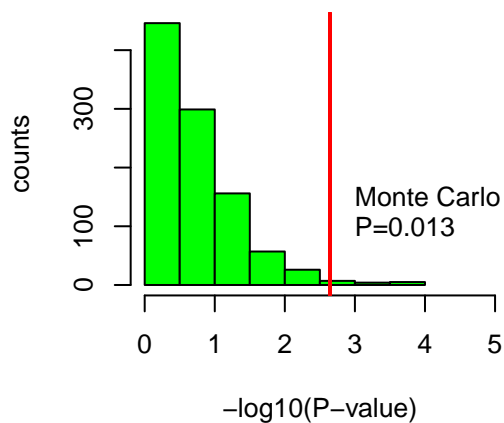

Supplement: Additional file 12 — Independent gene expression analysis of risk genes. (a) Average relative mRNA expression levels of 86 risk genes that could be mapped to the expression arrays of the integrated mRNA data set, comparing levels across 42 normal cervical samples (green) and 74 cervical cancers (red). The P-value is from a one-sided Wilcoxon rank sum test. (b) Corresponding t-statistics of differential expression (y-axis) of the 86 genes against -log10(P-value). The number of genes passing P = 0.05 threshold and that are over-/underexpressed in cancer are given. The P-value is from a binomial test assuming (32 + 14 = 46 trials) and under the null that there is an equal chance of under- or overexpression. (c) Comparison of the observed binomial test P-value in (b) (vertical red line) to those binomial test P-values obtained from 1,000 Monte Carlo runs (green histogram), in which 86 genes were selected at random from the integrated expression set. The P-value shows the fraction of runs which more extreme P-values than the observed one. This Monte Carlo analysis therefore corrects for any bias in assuming that there is an equal null probability of under- or overexpression. [file gm323-S12.PDF]

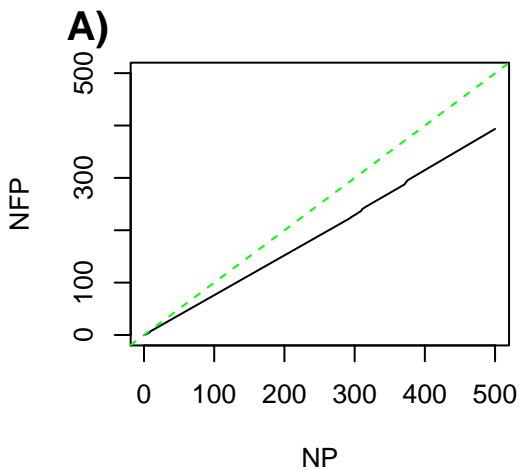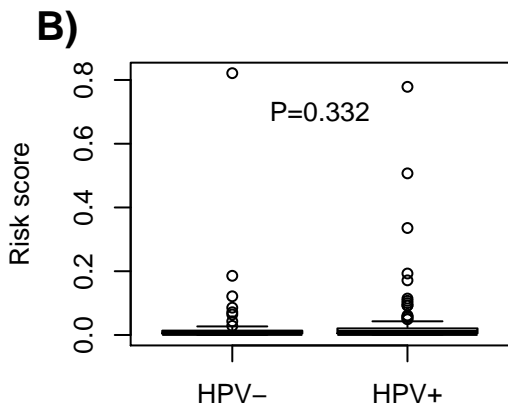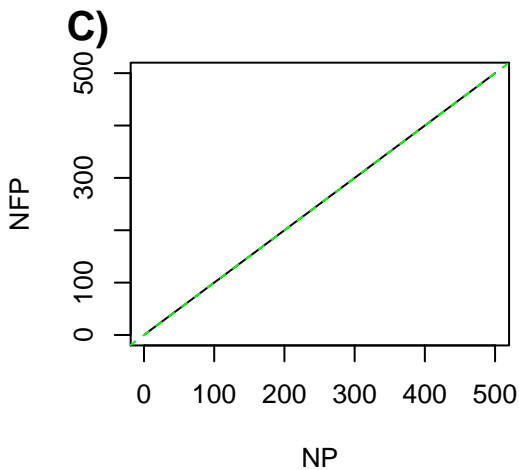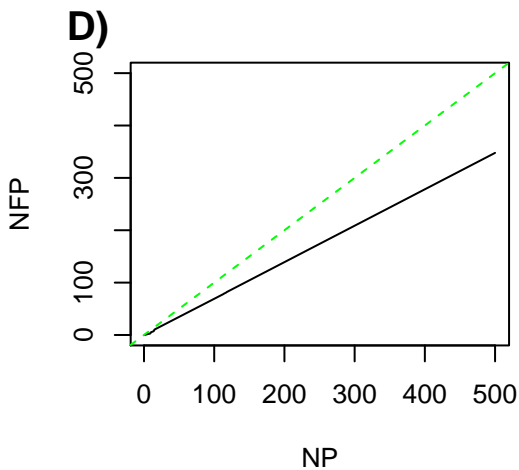

Supplement: Additional file 13 — DNA methylation and HPV status. (a) Expected number of false positives (NFP, y-axis) is plotted against the number of positives (NP, x-axis) for CpGs associated with HPV status using surrogate variable analysis (SVA) and all 152 samples in the ARTISTIC cohort. (b) Boxplot comparing the EVORA risk scores of the 152 samples against HPV status. The P-value is from a Wilcoxon rank sum test. (c) As (a), using SVA with HPV status as the phenotype but now only using the 77 samples that remained disease-free. (d) As (a), using SVA with HPV status as the phenotype but now only using the 75 samples that developed a CIN2+. In (a, c, d), the green dashed line indicates the null-line of no association. We note that even in (d) the association is very marginal since the FDR for the top 100 CpGs is over 50%. [file gm323-S13.PDF]

**A)**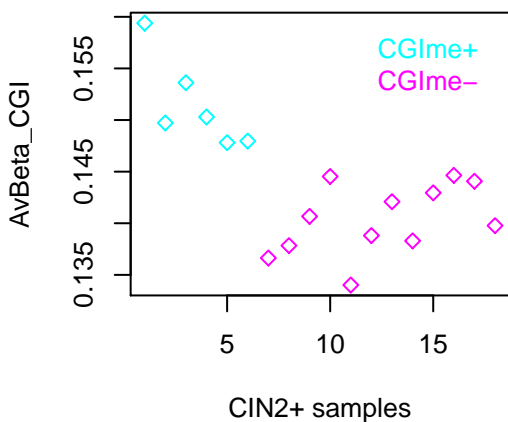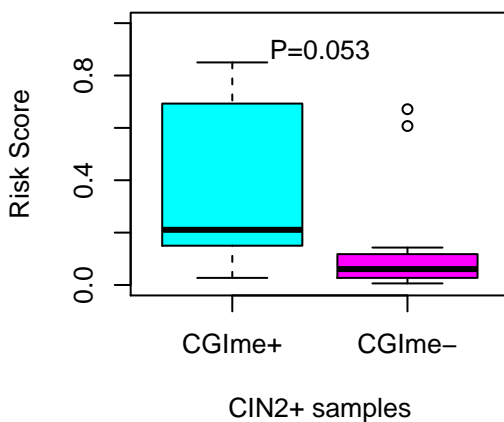**B)**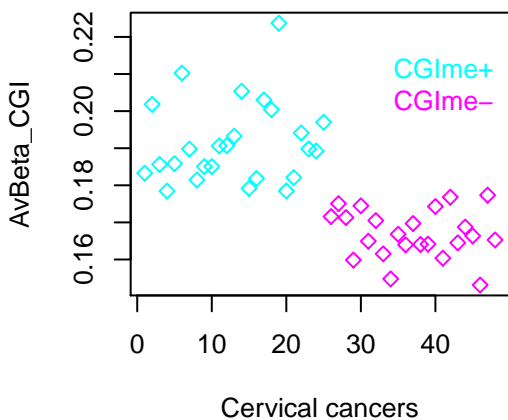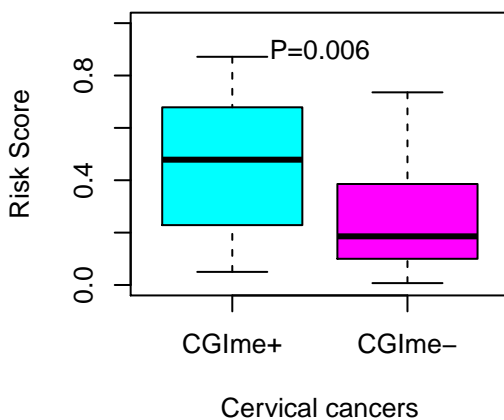

Supplement: Additional file 14 — Correlation of EVORA risk scores with CpG island methylation. (a) Left panel: average beta methylation level over all CpGs mapping to CpG islands (excluding the 140 risk CpGs) on the y-axis versus the CIN2+ sample index. A partitioning around medoids algorithm (pam from package cluster) was used to cluster the samples into two clusters of relative high and low methylation, and the samples have been ordered and colored accordingly. Right panel: boxplot of the EVORA risk scores defined over the 140 risk CpGs in the same set of CIN2+ samples, grouped according to the clustering in (a). The P-value is from a Wilcoxon rank sum test. (b) Exactly as (a), but now for the cervical cancer samples of set 2. [file gm323-S14.PDF]

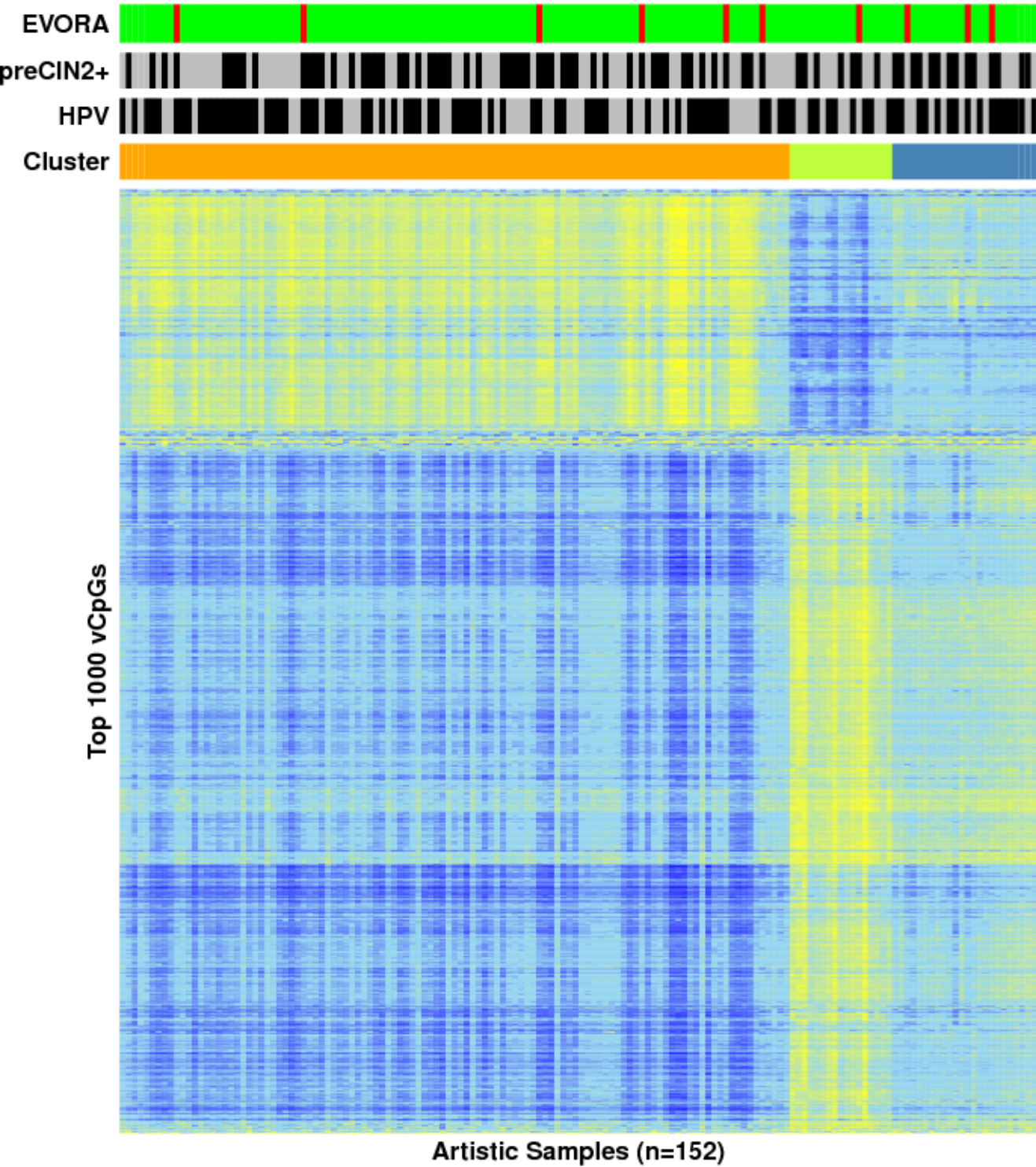

Supplement: Additional file 15 — Consensus clustering heatmap of the 152 ARTISTIC samples over the top 1,000 most variable CpGs (vCpGs). Color codes in the heatmap: yellow, beta < 0.3; skyblue, 0.3 < beta < 0.7; blue, beta > 0.7. The bars above the heatmap indicate the consensus cluster (three clusters were optimal), HPV status (black = HPV-positive, grey = HPV-negative), prospective CIN2+ status (black = prospective CIN2+, grey = control) and EVORA risk score (green = risk score < 0.1, red = risk score > 0.1). [file gm323-S15.PDF]

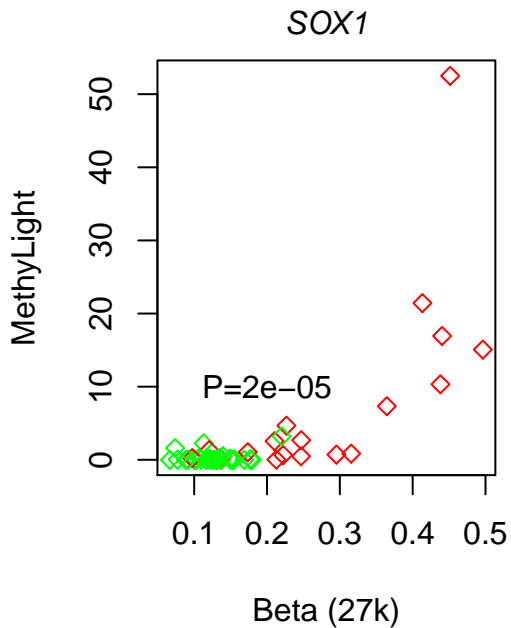

Supplement: Additional file 17 — Comparison of Methylight (PMR-value) based quantification of methylation (y-axis) with Infinium 27 K β-value (x-axis) for two of the identified risk genes (SOX1 and WT1) across the 48 LBC samples (set 1). The CpG on the 27 K array closest to the transcription start site and to the Methylight CpGs was used. The P-value is from a correlation test, testing the significance of the Spearman rank correlation. Red denotes the 18 CIN2+ samples, green denotes the 30 CIN2- samples. [file gm323-S17.PDF]
